# Supplementary material for: Unveiling the spatially confined oxidation processes in reactive electrochemical membranes
Source: Nat Commun. 2023 Oct 18;14:6590. doi: 10.1038/s41467-023-42224-3 (PMC10584896; doi:10.1038/s41467-023-42224-3)
Supplement: Supplementary file 1 — Supplementary Information [file 41467_2023_42224_MOESM1_ESM.pdf]

Supplementary Information

Unveiling the spatially confined oxidation processes in reactive electrochemical membranes

Yuyang Kang, Zhenao Gu*, Baiwen Ma, Wei Zhang, Jingqiu Sun, Xiaoyang Huang, Chengzhi Hu, Wonyong Choi, Jiuhui Qu*

*Corresponding authors: Zhenao Gu and Jiuhui Qu
Email: zagu@rcees.ac.cn (Z.G.), or jhqu@rcees.ac.cn (J.Q.)

This file includes:

Supplementary Methods
Supplementary Figures 1 to 38
Supplementary Tables 1 to 4
Supplementary References

Supplementary Methods

Chemicals and Materials

Sodium sulfate (Na_2SO_4, ≥99%), hydrochloric acid (HCl, 36%–38%), terephthalic acid (TA, ≥99%), formic acid (≥98%), potassium ferrocyanide trihydrate ($K_4Fe(CN)_6\cdot3H_2O$, ≥99.5%), potassium ferricyanide ($K_3Fe(CN)_6$, ≥99.5%), and sodium perchlorate monohydrate ($NaClO_4\cdot H_2O$, ≥99%) were purchased from Sinopharm (China); 4-chlorophenol (4-CP, ≥99%) and 2,4,6-trichlorophenol (≥98%) were purchased from Adamas (China). Tert-butyl alcohol (TBA, ≥99.5%), and 7-hydroxycoumarin (7-OH COU, ≥99%) were purchased from Aladdin (China); 2-hydroxyterephthalic acid (HTA, ≥97%) and 5,5-dimethyl-1-pyrroline N-oxide (DMPO, ≥97%) were purchased from Ark Pharm. Citric acid monohydrate (≥99.5%) was purchased from Psaitong (China). Coumarin (COU, ≥99%) and o-cresol (≥99%) were purchased from Tokyo Chemical Industry Co., Ltd. Acetonitrile (HPLC grade) and methanol (HPLC grade) were purchased from Fisher Scientific.

Analysis Methods

The concentrations of 4-CP and o-cresol were measured by high-performance liquid chromatography (HPLC, Agilent 1260) with a C18 column (4.6 × 250 mm, 5.0 μm), and the UV detector was set to be 281 nm. Isocratic elution was used with water and methanol (*v*/*v* = 40/60) as the mobile phase at a flow rate of 0.5 ml min^{-1}. TA was measured by HPLC at 265 nm with 0.01% aqueous formic acid and methanol (*v*/*v* = 40/60) as the mobile phase. HTA, the fluorescent adduct product of TA attacked by •OH, was analyzed by HPLC with a fluorescent detector at λ_{ex} = 315 nm and λ_{em} = 425 nm using the same mobile phase as TA. COU was measured by HPLC at 280 nm with 0.01% aqueous formic acid and methanol (*v*/*v* = 50/50) as the mobile phase. 7-OH COU, the fluorescent adduct product of COU attacked by •OH, was analyzed by HPLC with a fluorescent detector at λ_{ex} = 332 nm and λ_{em} = 471 nm using the same mobile phase as COU. In addition, 2,4,6-trichlorophenol was measured by HPLC at 295 nm with 0.01% aqueous formic acid and methanol (*v*/*v* = 20/80) as the mobile phase. Citric acid was measured by HPLC at 210 nm with 5 mM aqueous sulfuric acid and methanol (*v*/*v* = 96/4) as the mobile phase.

Energy Consumption Calculation

The mineralization current efficiency (MCE) for 4-CP degradation was calculated according to Supplementary equation (1)[1],

$$MCE = \frac{TOC_0 - TOC_t}{1.2 \times 10^4 xIt} nFV \times 100\% \quad (1)$$

where TOC_0 and TOC_t are the values of total organic carbon (TOC, mg C L^{-1}) at the beginning and time t, respectively. x is the number of carbon atoms in a 4-CP molecule, I is the applied current (A), n is the theoretical number of electrons for complete mineralization (estimate to be 26 for 4-CP), F is the Faraday constant (96,485 C mol^{-1}), and V is the volume of the solution (L).

The total energy consumption was calculated as the sum of electrical energy (E_E, kWh m^{-3}) and pumping energy (E_P) normalized per log removal of 4-CP. The E_E value was calculated according to Supplementary equation (2)[2],

$$E_{\mathrm{E}} = 10^{-3} \times \frac{V_{\mathrm{cell}} I}{Q} \tag{2}$$

where V_{cell} is the cell potential (V), I is the current used in the experiment (i.e., 2.54 cm^2 × 19.7 mA cm^{-2} × 10^{-3} = 0.05 A), Q is the volumetric flow rate at which 90% 4-CP removal was achieved (m^3 h^{-1}).

The E_P value was calculated by Supplementary equation (3)[2],

$$E_{\mathrm{P}} = 3.6 \times 10^{-5} \times \frac{\rho g \Delta P}{\eta} \tag{3}$$

where ρ is the density of water (997 kg m^{-3}), g is the gravitational constant (9.81 m s^{-2}), ΔP is the transmembrane pressure at a given flow rate (bar), and η is the pump efficiency (assumed as 0.7).

Finite Element Simulation

The finite element analysis was simulated using COMSOL Multiphysics 5.3a. Microchannels with diameters of 7.3 μm, 17.5 μm, 45.0 μm, and 105.0 μm were modeled, respectively. The channel length was set to be 3 mm, which is the same as the thickness of REMs. The numbers of channels were estimated to be 239, 56, 12, and 3 for the four diameters, respectively, based on the porosity of REMs (Supplementary Table 3), which is a significant factor that influence the surface potential distribution in porous electrode[3].

The 3D geometry "CFD" module was used to solve the flow velocity and streamline distribution in the microchannels. The solution was distributed into reactive channels in the inlet region and get degraded in the zone near the channel surface (Supplementary Fig. 26). The range of hydraulic residence time (HRT) in the simulation (2.7–54.5 s) was consistent with the experiment (2.7–54.5 s). The "transport of diluted species" module was used to solve the concentration distribution. The mathematical model of the mass transfer process includes Fick's law, the convection process, and the reaction process. As we mainly simulated the mass transfer process of neutral molecules inside the anode and 0.33 M $NaClO_4$ electrolyte was used during experiments, the electrostatic migration

term was not included in the model[4]. As the current density is influenced by the depth of channel[5,6], the "secondary current distribution" module was used to investigate the current distribution ($\mathbf{j}(x)$, A m^{-2}) in the flow-through system. The Butler-Volmer equation was used to solve for the current distribution (Supplementary equation (4))[7],

$$i = FAk^0\left[e^{-\alpha f(E-E^0)} - e^{(1-\alpha)f(E-E^0)}\right] \tag{4}$$

where i is the local current (A), F is the faraday constant (C mol^{-1}), A is the surface area of electrode (m^2), k^0 is the standard rate constant (m s^{-1}), α is the transfer coefficient, $f = \frac{F}{RT}$, E^0 is the formal potential (V), and E is the electrode potential (V).

The applied current (i_{app}, A) (Fig. 4b) was determined according to the porosity and current in the experiment (Supplementary equation (5)),

$$i_{app} = I\frac{S}{pA} \tag{5}$$

where I is the current in the experiment (0.05 A unless otherwise mentioned), p is the porosity of REM, A is the geometry surface area of the electrode (2.54×10^{-4} m^2), and S (m^2) is the cross-section area of the simulated channel.

The inner surface of channels was defined as the reaction surface. For simplicity, the generation of •OH in the region with a potential lower than 2.8 V_{RHE} was ignored[8,9]. In the region with a potential higher than 2.8 V_{RHE}, the production of •OH ($r_{\mathrm{g,\bullet OH}}$, mol m^{-2} s^{-1}) is expressed in terms of current density by Supplementary equation (6)[4,10],

$$r_{\mathrm{g,\bullet OH}} = k_{\mathrm{g,\bullet OH}}\frac{j(x)}{F} \tag{6}$$

where $k_{g,\bullet OH}$ (dimensionless) was estimated using a TA degradation experiment (Supplementary Fig. 34), $j(x)$ is the local current density (A m^{-2}).

Similar to the •OH production, a surface reaction kinetic equation was used to model the direct electron transfer (DET) reaction and the flux of reactants at the electrode surface[11] (Supplementary equation (7)),

$$r_{\mathrm{4-CP,DET}} = k_{\mathrm{f,4-CP}}N_{\mathrm{D}}c_{\mathrm{4-CP}} = k_{\mathrm{4-CP,DET}}\frac{j(x)}{F}c_{\mathrm{4-CP}} \tag{7}$$

Where $k_{\mathrm{f,4-CP}}$ represents the forward rate constant of 4-CP DET reaction, N_{D} is the number of reaction sites per unit of surface area, and $c_{\mathrm{4-CP}}$ is the concentration of 4-CP in the electrode surface. Additionally, $k_{\mathrm{4-CP,DET}}$, the rate constant (m^3 mol^{-1}), was optimized in the simulation.

Here it is assumed that the DET reaction does not compete for active sites with the production of •OH and the DET reaction is chemically irreversible[11]. It is also assumed that the number of surface sites for the DET reaction is directly proportional to the current density.

Other reactions were defined as follows:

No.	Reactions	Rate constants
1	$\bullet OH + 4 - CP \rightarrow 4 - CP_{products}$	$k_{\text{4-CP, •OH}}$
2	$\bullet OH + \bullet OH \rightarrow H_2O_2$	$k_{\text{•OH, •OH}}$
3	$\bullet OH + 4 - CP_{products} \rightarrow 4 - CP_{products}$	$k_{\text{4-CP products, •OH}}$

The mineralization of most organic substances is a multi-step oxidation process and involves a large quantity of •OH[12-14]. For this reason, the intermediate products are set to be accumulated and would not be consumed in the whole oxidation process.

The simulation of HTA production was conducted in a similar manner, where the same HRT as the experiment (2.7 s) was used. The kinetic constant was set to be 4.4×10^{6} m^3 s^{-1} mol^{-1} [8]. The value of the percent yield was set to be 8.8% (Supplementary Fig. 28). DET reaction was not taken into consideration in the simulation.

All parameters and values used in the simulation are shown in Supplementary Table 4. When the simulation was performed at different parameter values (e.g., pore size and applied current), all other parameters remained unchanged.

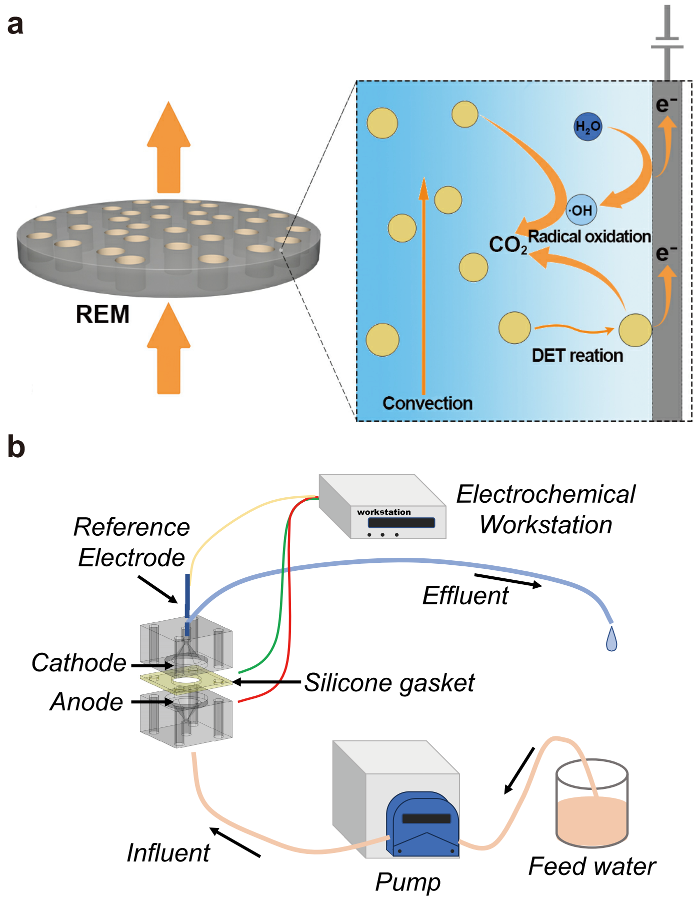

Supplementary Fig. 1. Schematics of the REMs and single-pass filtration system. a Schematic of the reaction process in the microchannels. **b** The dead-end filtration assembly used to conduct all the electrochemical experiments, where the membrane flux was controlled by a peristaltic pump. The thickness of REMs is 3 mm. The gasket possesses a circular hole with a diameter of 18 mm to allow for the water flow.

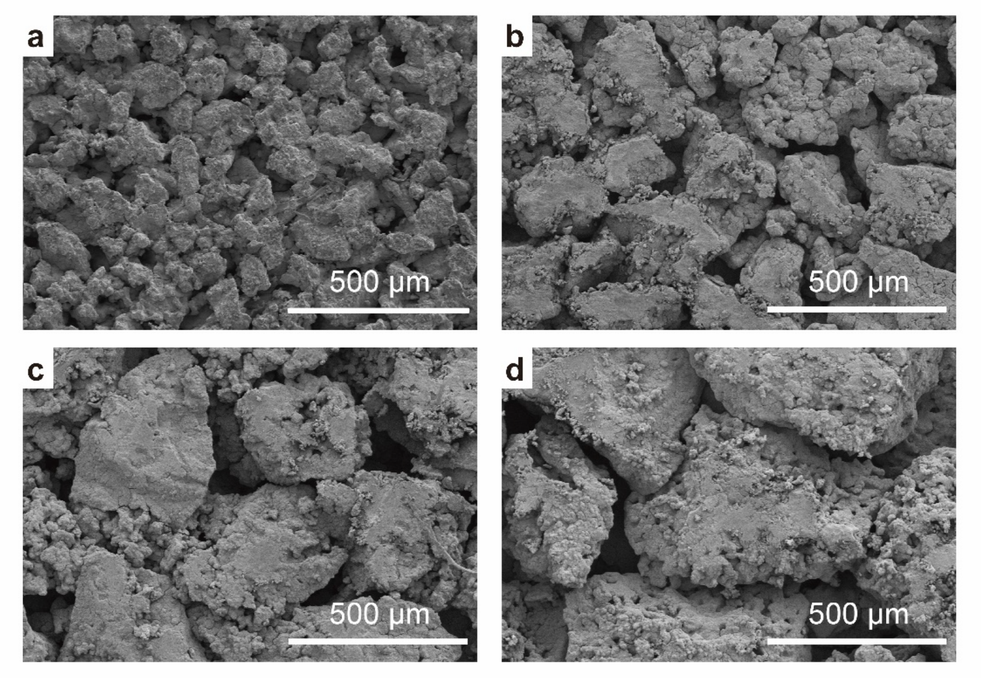

Supplementary Fig. 2. Macroscopic pores in REMs. a-d SEM images (top view) of (**a**) REM$_{7\mu m}$, (**b**) REM$_{17\mu m}$, (**c**) REM$_{45\mu m}$, and (**d**) REM$_{105\mu m}$.

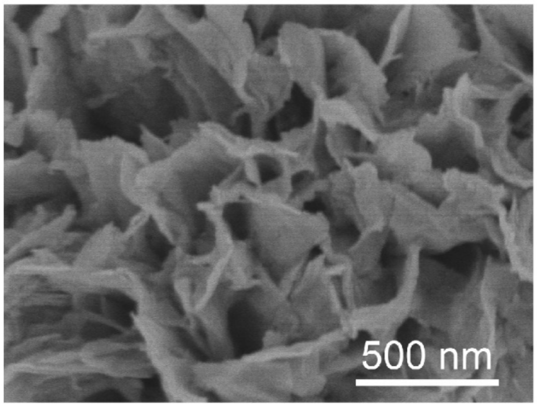

Supplementary Fig. 3. Enlarged SEM image of REMs. TiO_2 nanosheets with a length of 0.5 μm and a thickness of approximately 20 nm were densely grown on Ti substrates.

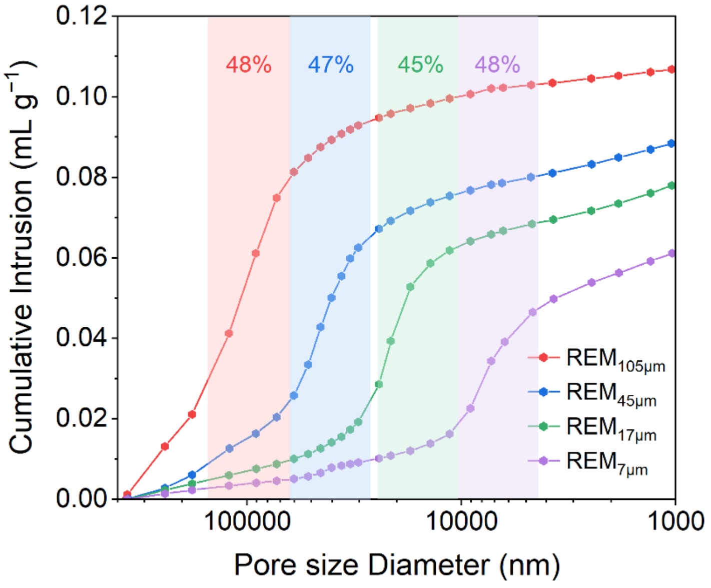

Supplementary Fig. 4. The pore size distribution of REMs by mercury intrusion method. The predominant pore sizes in the REMs were 7, 17, 45, and 90 – 120 μm, respectively. These electrodes are denoted as $REM_{7μm}$, $REM_{17μm}$, $REM_{45μm}$, and $REM_{105μm}$, respectively. Note that $REM_{105μm}$ was denoted using the arithmetic mean of the two predominant pore sizes. Over 45% of the total pore volume falls within the ±40% range around the predominant size, indicating that the selected pore sizes are representative.

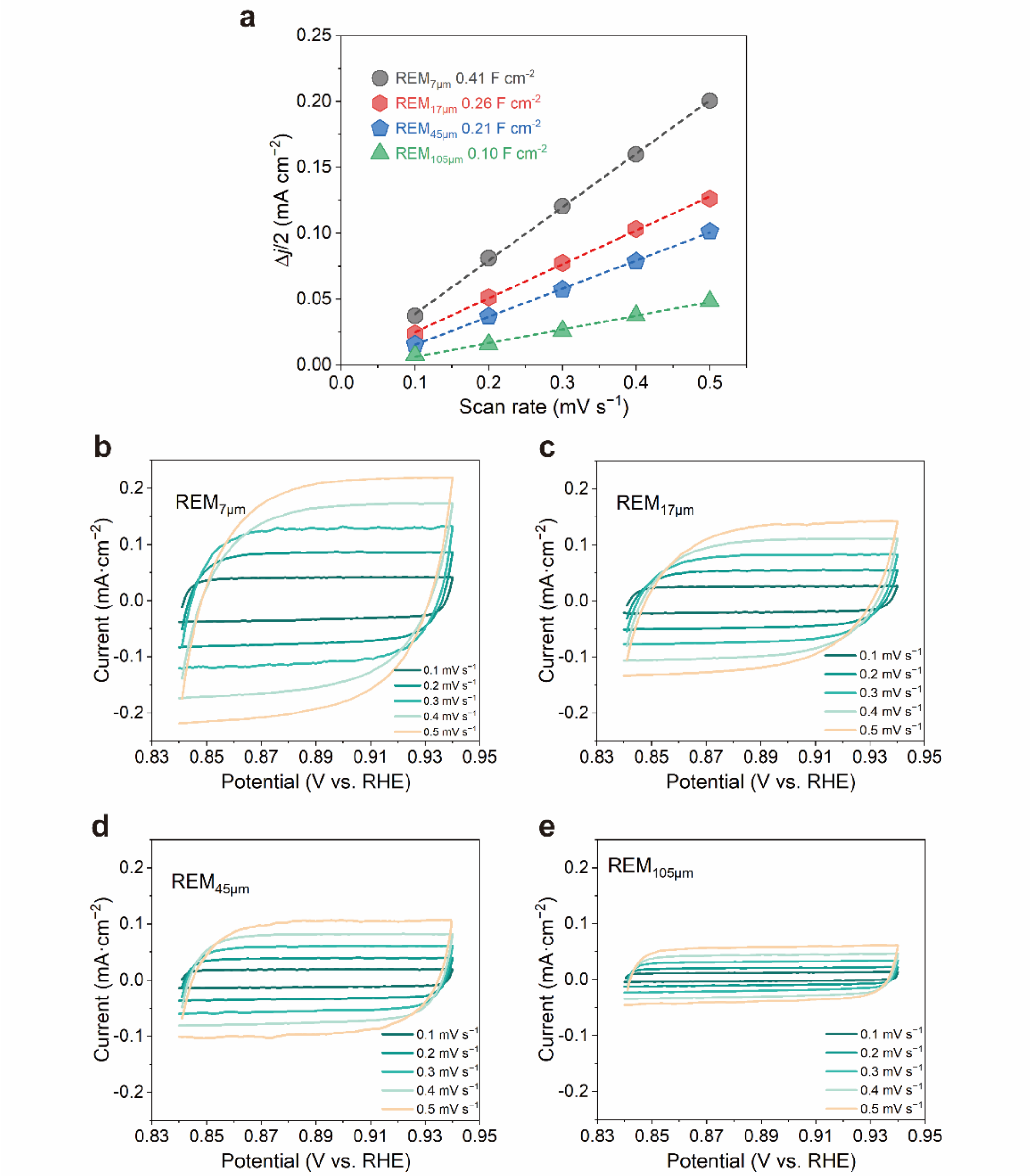

Supplementary Fig. 5. The electrical double-layer capacitance (C_{dl}) and corresponding cyclic voltammetry (CV) curves of different REMs. a C_{dl} by plotting current variation against the scan rate to fit a linear regression. **b-e** Cyclic voltammetry (CV) curves of (**b**) $REM_{7μm}$, (**c**) $REM_{17μm}$, (**d**) $REM_{45μm}$, and (**e**) $REM_{105μm}$. C_{dl} values were measured at the potential of 0.89 V vs. RHE. Electrolyte: 0.33 M $NaClO_4$. Note that C_{dl} value is proportional to the electrochemically active surface area. This result is well consistent with the Brunauer–Emmett–Teller (BET) surface area (Supplementary Table 1), which was used for the analysis in this study.

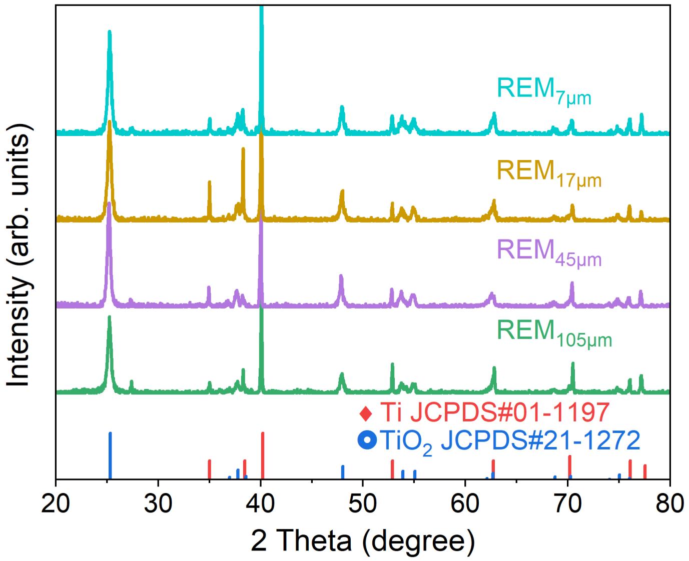

Supplementary Fig. 6. XRD patterns of the REMs. The diffraction peaks at 35.0°, 38.4°, and 40.2° correspond to the metallic Ti substrate (JCPDS#01-1197)[15]. The distinct peaks at 25.3° and 48.0° can be well indexed to the (101) and (200) planes of anatase TiO_2 (JCPDS#21-1272)[16], respectively.

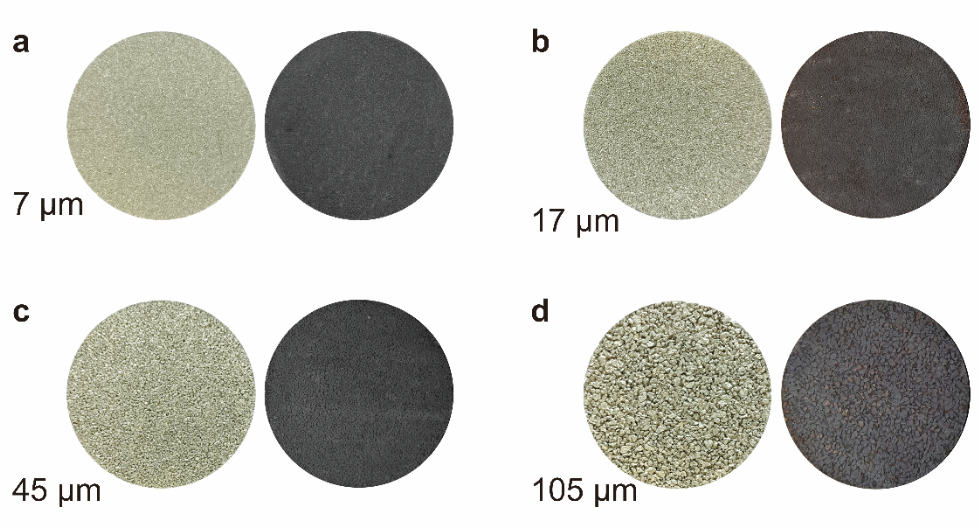

Supplementary Fig. 7. Optical photographs of Ti substrates (left) and the corresponding REMs (right). **a** $REM_{7\mu m}$, **b** $REM_{17\mu m}$, **c** $REM_{45\mu m}$, and **d** $REM_{105\mu m}$.

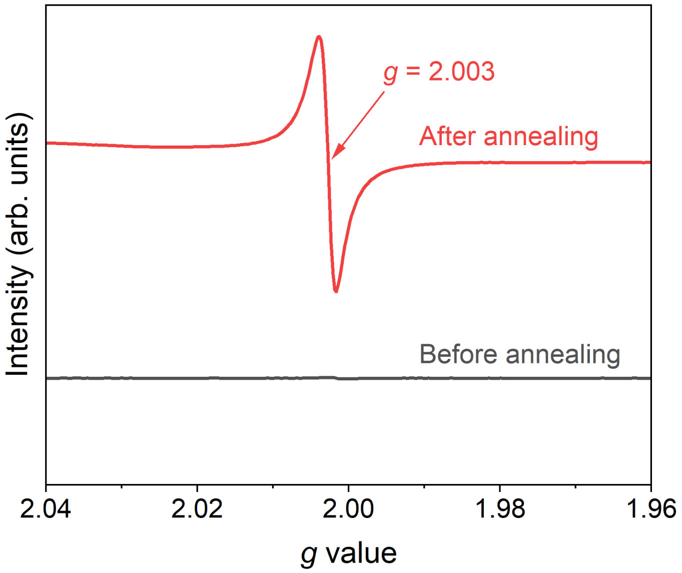

Supplementary Fig. 8. ESR spectra of $REM_{7\mu m}$ before and after annealing in the argon atmosphere. The signal at $g = 2.003$ is a typical sign of oxygen vacancies[16].

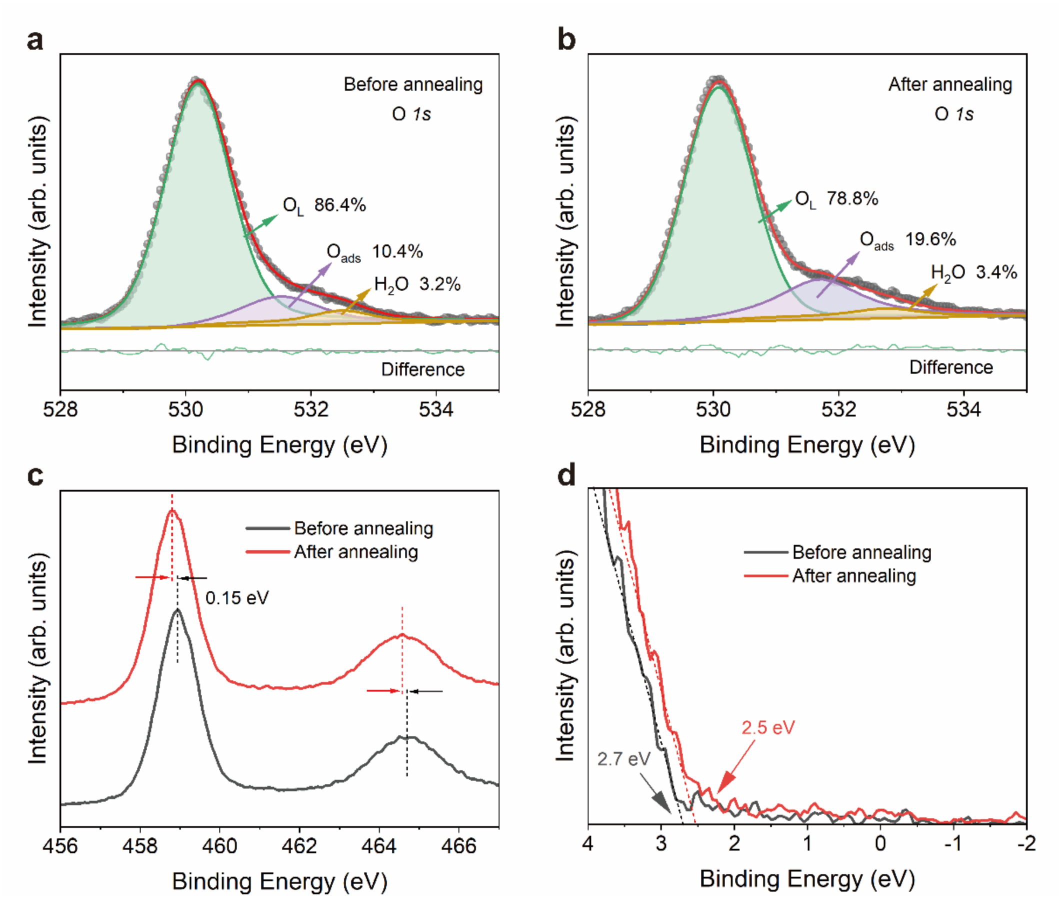

Supplementary Fig. 9. XPS spectra of REM$_{7\mu m}$ before and after annealing in an argon atmosphere. a, b O *1s* XPS spectra of REM$_{7\mu m}$ before (**a**) and after (**b**) annealing in an argon atmosphere. **c** Ti *2p* XPS spectra. **d** valence band XPS spectra. For the O *1s* spectra, the peaks at 530.3, 531.5, and 532.6 eV were attributed to lattice oxygen (O$_L$), adsorbed oxygen (O$_{ads}$), and surface oxygen (H_2O), respectively[17,18]. The increase of the O$_{ads}$ demonstrates the existence of oxygen vacancies accompanied by localized electrons richness[19]. The Ti *2p* peak shifted to a lower binding energy by −0.15 eV, indicating the lattice Ti^{4+} atoms were partly reduced to Ti^{3+}. Consistent with the deconvolution of the O *1s* XPS band, the unsaturated Ti^{3+} further suggested the existence of oxygen vacancies[20]. The position of the valance band edge shifted from 2.70 eV to 2.50 eV, showing a narrowed band gap after the thermal treatment[21].

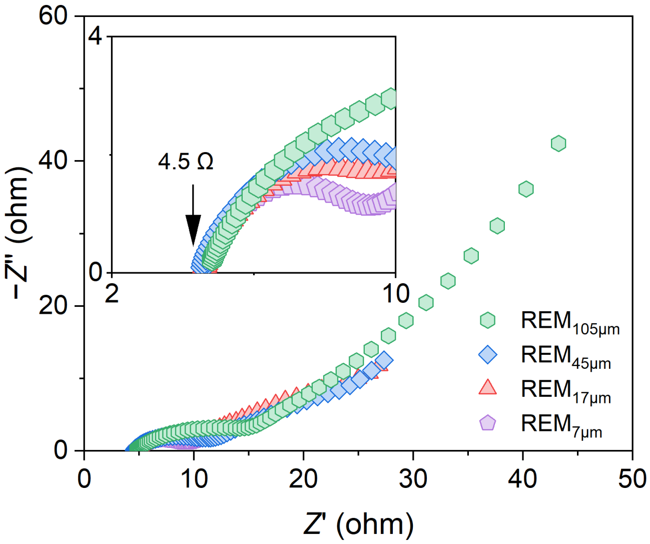

Supplementary Fig. 10. Nyquist plots of REMs. The data was obtained at 0.61 V_{RHE} in 0.33 M $NaClO_4$ (pH = 7).

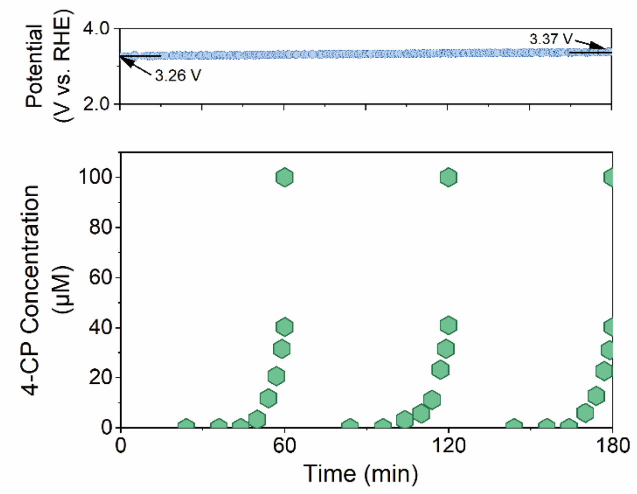

Supplementary Fig. 11. Potential-time curve and 4-CP degradation performance of $REM_{7\mu m}$. Current density: 19.7 mA cm^{-2}. Electrolyte: 0.33 M $NaClO_4$. All prepared electrodes were subjected to pre-electrolysis for 3 hours. Subsequently, all experiments on each electrode were performed within 180 minutes after the pre-electrolysis.

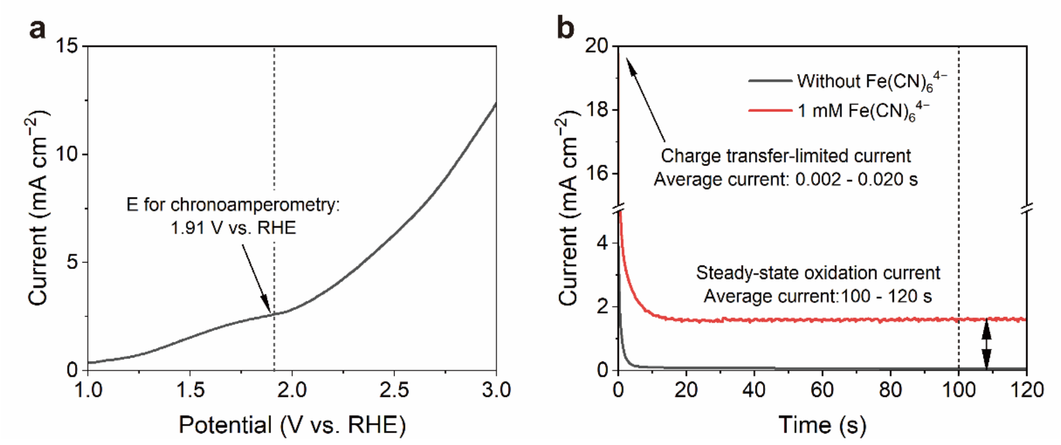

Supplementary Fig. 12. Determination of oxidation current and charge transfer-limited current. **a** Representative linear sweep voltammetry (LSV) curve of $REM_{105 \mu m}$ for $Fe(CN)_6^{4-}$ oxidation. **b** Representative chronoamperometry curves to derive the charge transfer-limited current and steady-state oxidation current of $Fe(CN)_6^{4-}$ [22]. LSV measurements were conducted at a membrane flux of 0.11×10^{-2} m s^{-1} in an electrolyte containing 1 mM $Fe(CN)_6^{4-}$, 2 mM $Fe(CN)_6^{3-}$, and 0.33 M $NaClO_4$, which was briefly denoted as 1 mM $Fe(CN)_6^{4-}$. A potential of 1.91 V_{RHE} was selected to avoid the side reactions (i.e., oxidation of water molecules). The average oxidation current at the initial phase (0.002 – 0.020 s) was calculated as the charge-transfer limited current. Meanwhile, the current at a steady state (100 – 120 s) was employed as the mass-transfer limited current.

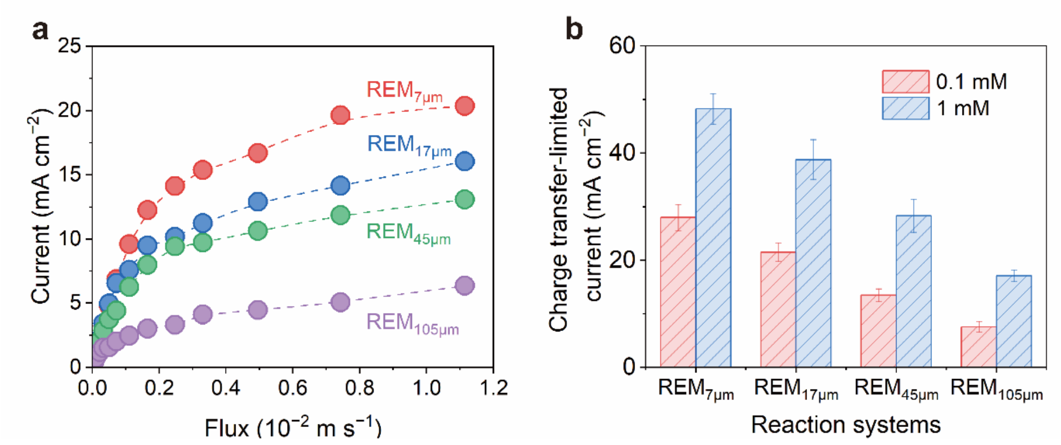

Supplementary Fig. 13. Oxidation current and charge transfer-limited current of $Fe(CN)_6^{4-}$ on REMs. a Steady-state (mass transfer-limited) oxidation current of $Fe(CN)_6^{4-}$ on REMs at different fluxes. **b** Charge transfer-limited current on REMs at different concentrations of $Fe(CN)_6^{4-}$. Error bars represent the standard deviations from the data obtained in 0.002 – 0.020 s. The experiments in panel a were conducted at 1.91 V_{RHE} electrode potential in an electrolyte containing 1 mM $Fe(CN)_6^{4-}$, 2 mM $Fe(CN)_6^{3-}$, and 0.33 M $NaClO_4$ (denoted as 1 mM in panel b). The charge transfer-limited currents were also collected in an electrolyte containing 0.1 mM $Fe(CN)_6^{4-}$, 0.2 mM $Fe(CN)_6^{3-}$, and 0.33 M $NaClO_4$ (denoted as 0.1 mM in panel b). The charge-transfer limited current was determined according to Supplementary Fig. 12b. The charge-transfer limited current was not linearly related to the electroactive area of REMs when 1 mM $Fe(CN)_6^{4-}$ was used. This can be attributed to the electrical resistance (Supplementary Fig. 10), and nonuniform potential distribution (Fig. 4b) in the experimental system, which affects the charge transfer in REMs. In the presence of 1 mM $Fe(CN)_6^{4-}$, the steady-state oxidation currents on REMs at relatively high flux are comparable to the corresponding charge transfer-limited currents. For instance, the oxidation current on $REM_{7μm}$ reached 20.4 mA cm^{-2} at a flux of 1.1×10^{-2} m s^{-1}, which is comparable to the charge transfer limit (48.3 mA cm^{-2}). Under such a condition, the oxidation of $Fe(CN)_6^{4-}$ is controlled by both the charge transfer limitation and mass transport. Meanwhile, the oxidation currents on REMs in 0.1 mM $Fe(CN)_6^{4-}$ are much smaller than the respective charge transfer-limited currents, as shown in Supplementary Fig. 13b and Fig. 2a in the main text. Therefore, an electrolyte with 0.1 mM $Fe(CN)_6^{4-}$ was used to further analyze the mass transport mechanism in micropores.

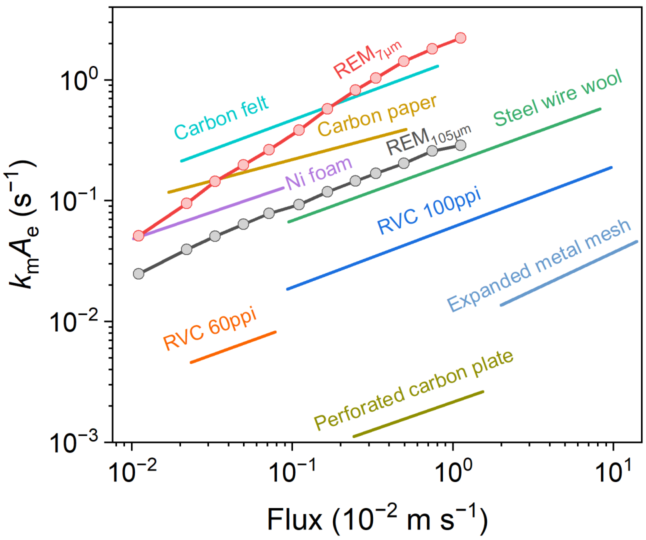

Supplementary Fig. 14. Relationship between k_mA_e and flux for various REMs[23]. The performance of mass transfer on $REM_{105μm}$ is superior to reticulated vitreous carbon (RVC) and expanded metal mesh. Further reducing the pore size to 7 μm resulted in improved mass transfer capability that is higher than the carbon fiber materials.

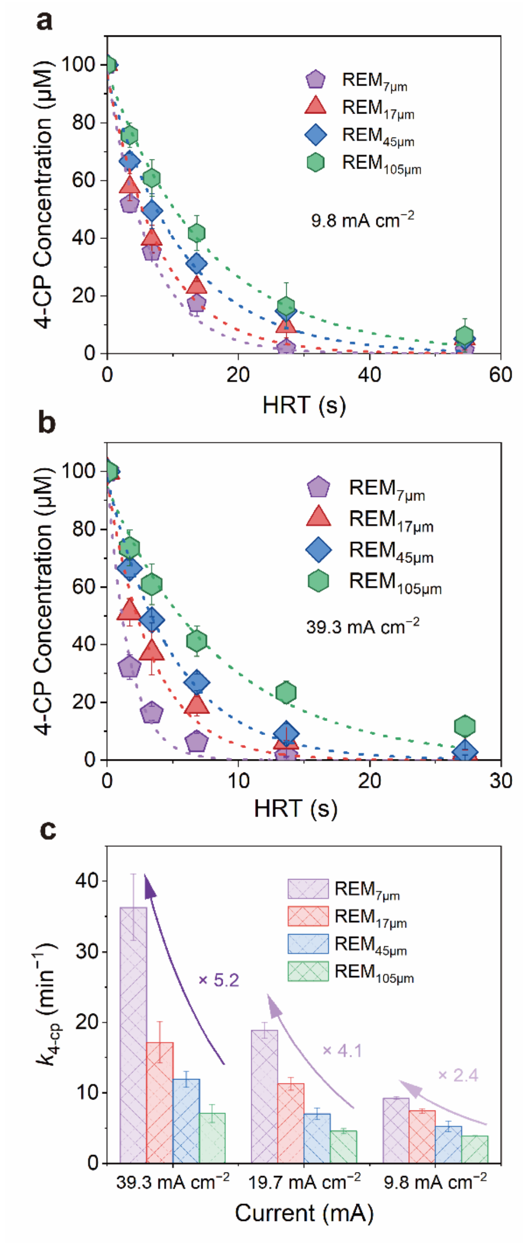

Supplementary Fig. 15. The 4-CP degradation performance on REMs at current densities of 9.8 mA cm^{-2} and 39.3 mA cm^{-2}. a The effluent 4-CP concentration as a function of HRT at a current density of 9.8 mA cm^{-2}. **b** The effluent 4-CP concentration as a function of HRT at a current density of 39.3 mA cm^{-2}. **c** The comparison of pseudo-first-order kinetic constants on REMs at different current densities. Error bars represent the data from duplicate tests.

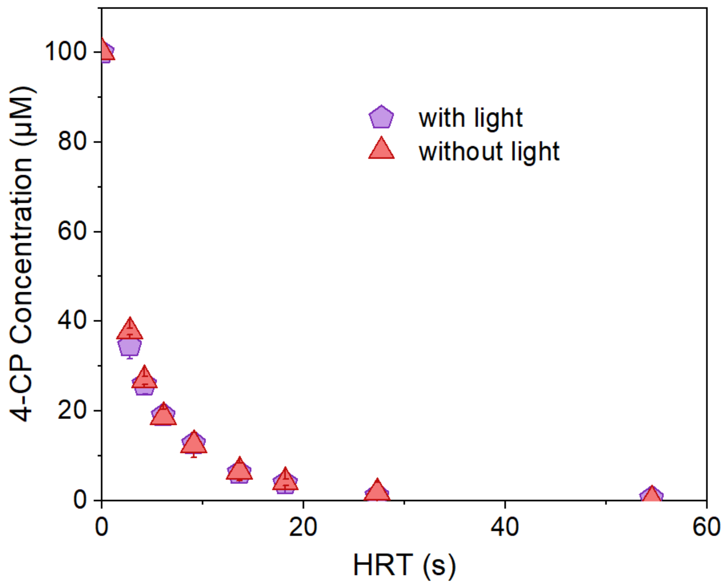

Supplementary Fig. 16. Degradation performance of 4-CP on $REM_{7\mu m}$ under room light and dark conditions. Current: 19.7 mA cm^{-2}. Reaction area: 2.54 cm^2. pH = 7. Error bars represent the data from duplicate tests.

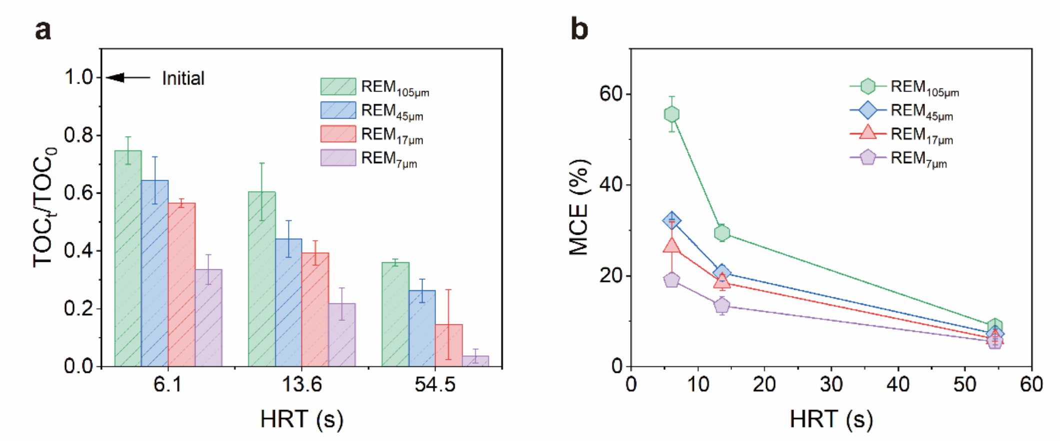

Supplementary Fig. 17. TOC removal performance and corresponding MCE values on REMs at different HRTs. a The TOC removal performance on REMs at different HRTs. **b** The corresponding MCE. MCE is calculated assuming that 4-CP is mineralization to CO_2 and H_2O. Error bars represent the data from duplicate tests.

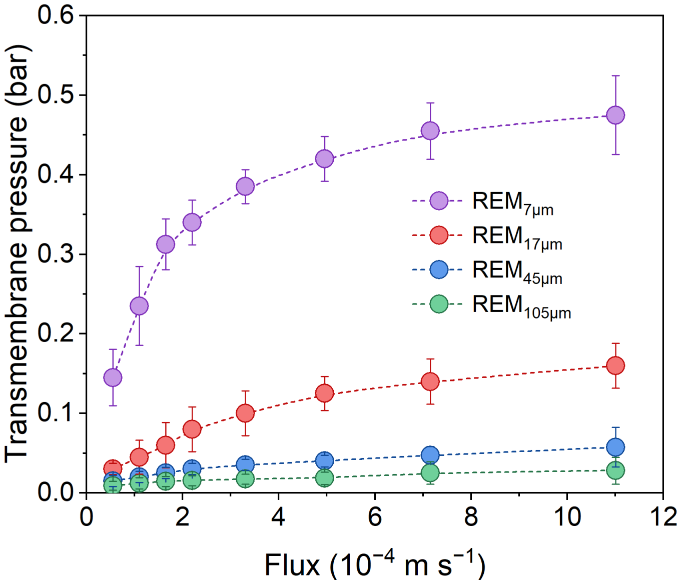

Supplementary Fig. 18. Transmembrane pressure as a function of flux.

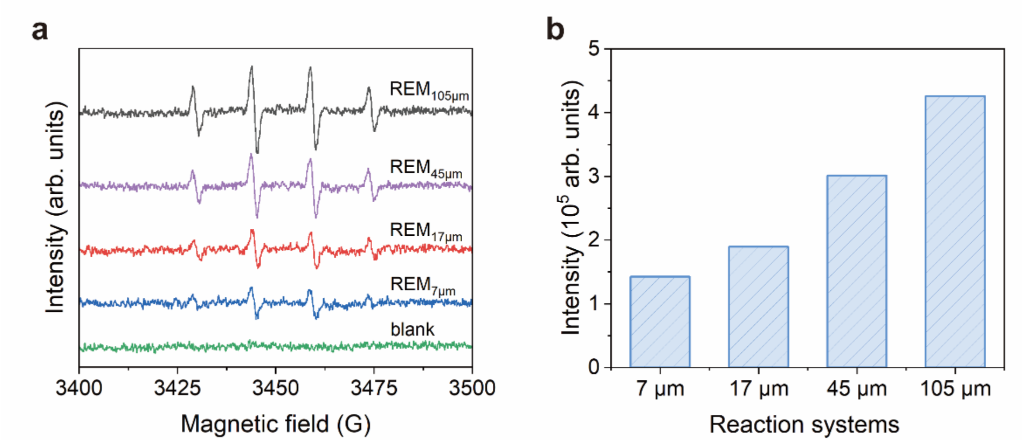

Supplementary Fig. 19. DMPO spin trapping ESR spectra of REMs. a The ESR spectra of the REMs. **b** Peak intensity for DMPO-•OH on the REMs. The experiments were performed in the same conditions as the degradation experiment, except that 4-CP was absent in the electrolyte. The quartet-signal with the intensity of 1:2:2:1 can be attributed to DMPO-•OH[24]. The experiments were performed at an HRT of 2.7 s.

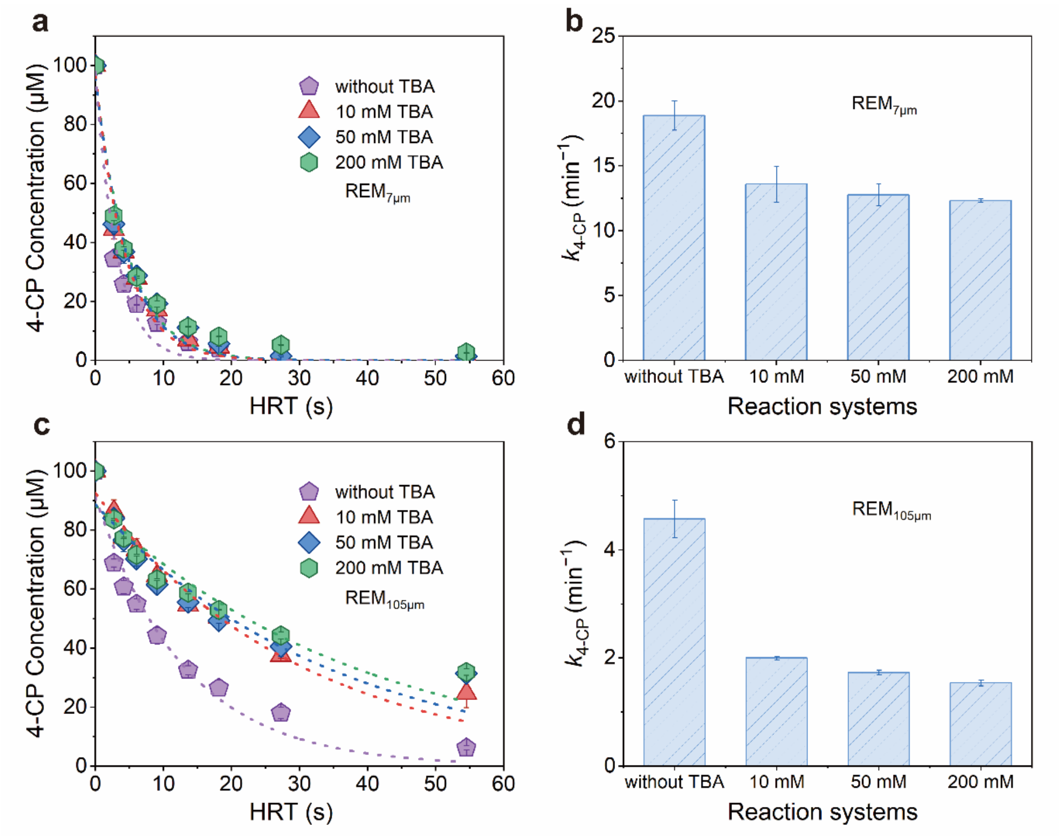

Supplementary Fig. 20. The effluent 4-CP concentration as a function of HRT at different TBA concentrations. a The effluent 4-CP concentration as a function of HRT on $REM_{7μm}$. **b** The corresponding pseudo-first-order kinetic constants on $REM_{7μm}$. **c** The effluent 4-CP concentration as a function of HRT on $REM_{105μm}$. **d** The corresponding pseudo-first-order kinetic constants. The dashed lines represent the fitted degradation curves. The initial concentration of organics: 100 μM. Current: 19.7 mA cm^{-2}. Error bars represent the data from duplicate tests.

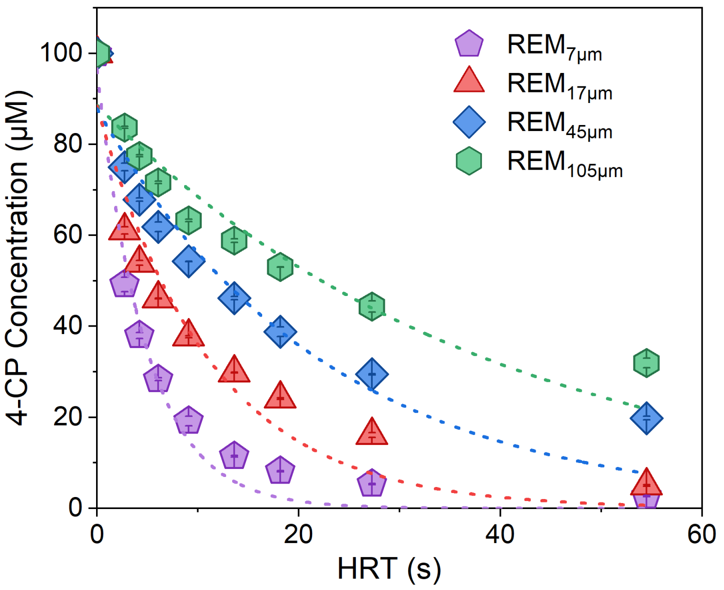

Supplementary Fig. 21. The effluent 4-CP concentration as a function of HRT in different REMs in the presence of TBA. The dashed lines represent the fitted degradation curves. The initial concentration of organics: 100 μM. TBA concentration: 200 mM. Current: 19.7 mA cm^{-2}. Error bars represent the data from duplicate tests.

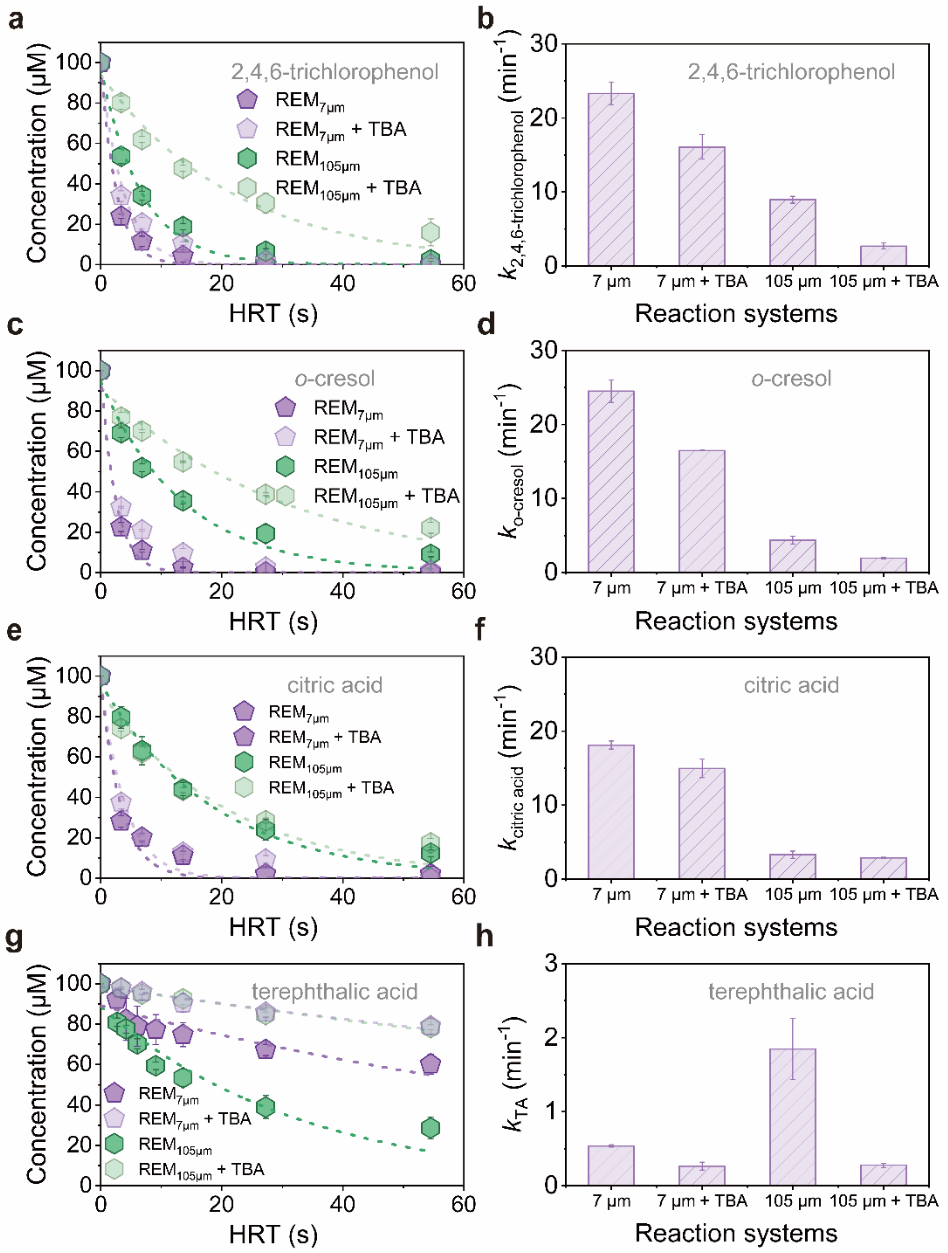

Supplementary Fig. 22. Performance for electrochemical oxidation of organics on REMs. a The effluent 2,4,6-trichlorophenol concentration as a function of HRT. **b** The pseudo-first-order kinetic constants of 2,4,6-trichlorophenol degradation. **c** The effluent *o*-cresol concentration as a function of HRT. **d** The pseudo-first-order kinetic constants of *o*-cresol degradation. **e** The effluent citric acid concentration as a function of HRT. **f** The pseudo-first-order kinetic constants of citric acid degradation. **g** The effluent TA concentration as a function of HRT. **h** The pseudo-first-order kinetic constants of TA degradation. The dashed lines represent the fitted degradation curves. The initial concentration of organics: 100 μM. Current: 19.7 mA cm^{-2}. Error bars represent the data from duplicate tests.

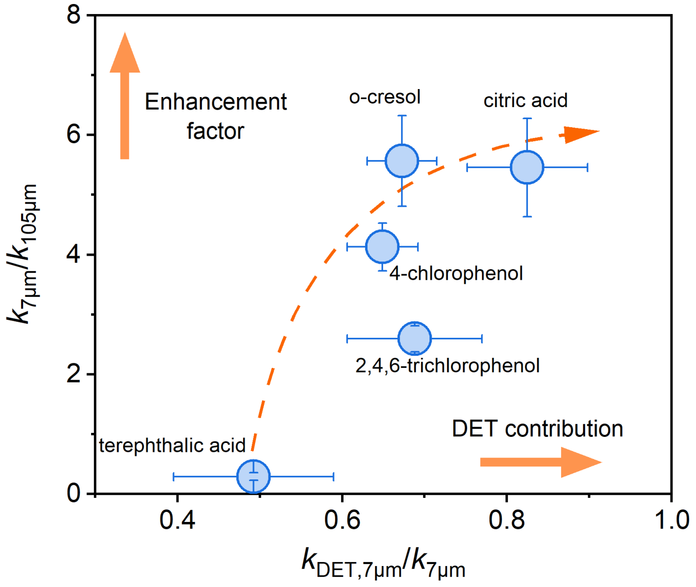

Supplementary Fig. 23. Relationship between enhancement factors and the DET contribution in REM$_{7μm}$ of different model reactants. Error bars represent the data from duplicate tests.

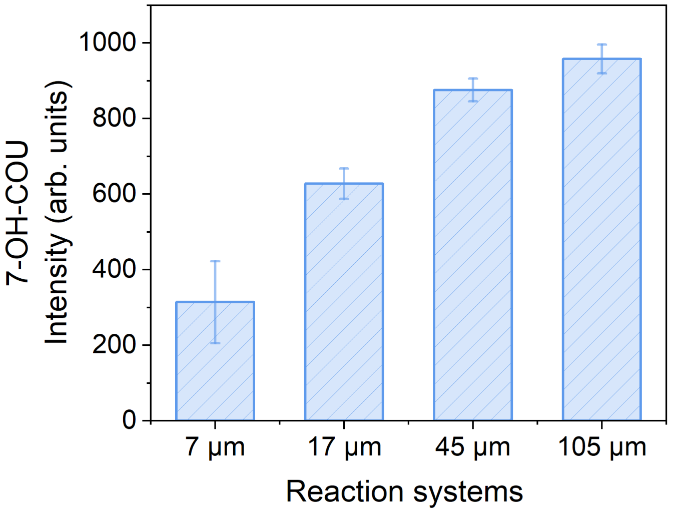

Supplementary Fig. 24. The production of 7-OH COU. The initial concentration of organics: 100 μM. Current: 19.7 mA cm^{-2}. HRT: 2.7 s. Error bars represent the data from duplicate tests.

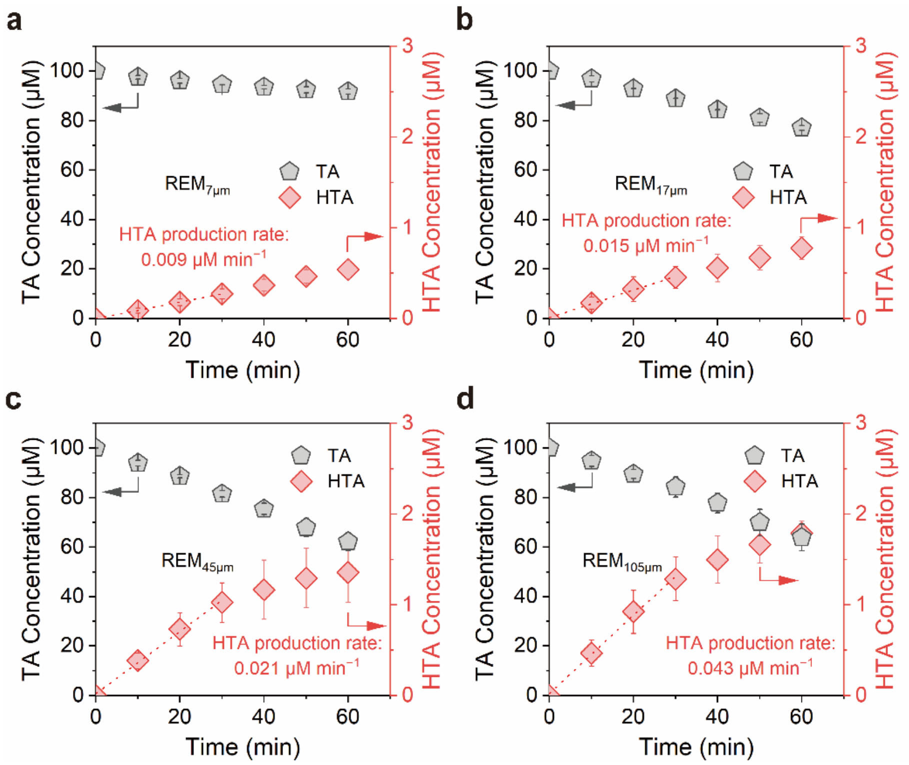

Supplementary Fig. 25. The production of •OH in a recirculation mode. a-d The decay of TA and production of HTA in the recirculation mode on (**a**) $REM_{7\mu m}$, (**b**) $REM_{17\mu m}$, (**c**) $REM_{45\mu m}$, and (**d**) $REM_{105\mu m}$. The initial concentration of TA: 100 μM. Current: 19.7 mA cm^{-2}. Volume of water sample: 300 mL. Electrolyte: 0.33 M $NaClO_4$. Flux: 0.066 mL cm^{-2} s^{-1}. The dotted line represents the linear regression curve obtained from the data in the initial 30 min. Error bars represent the data from duplicate tests.

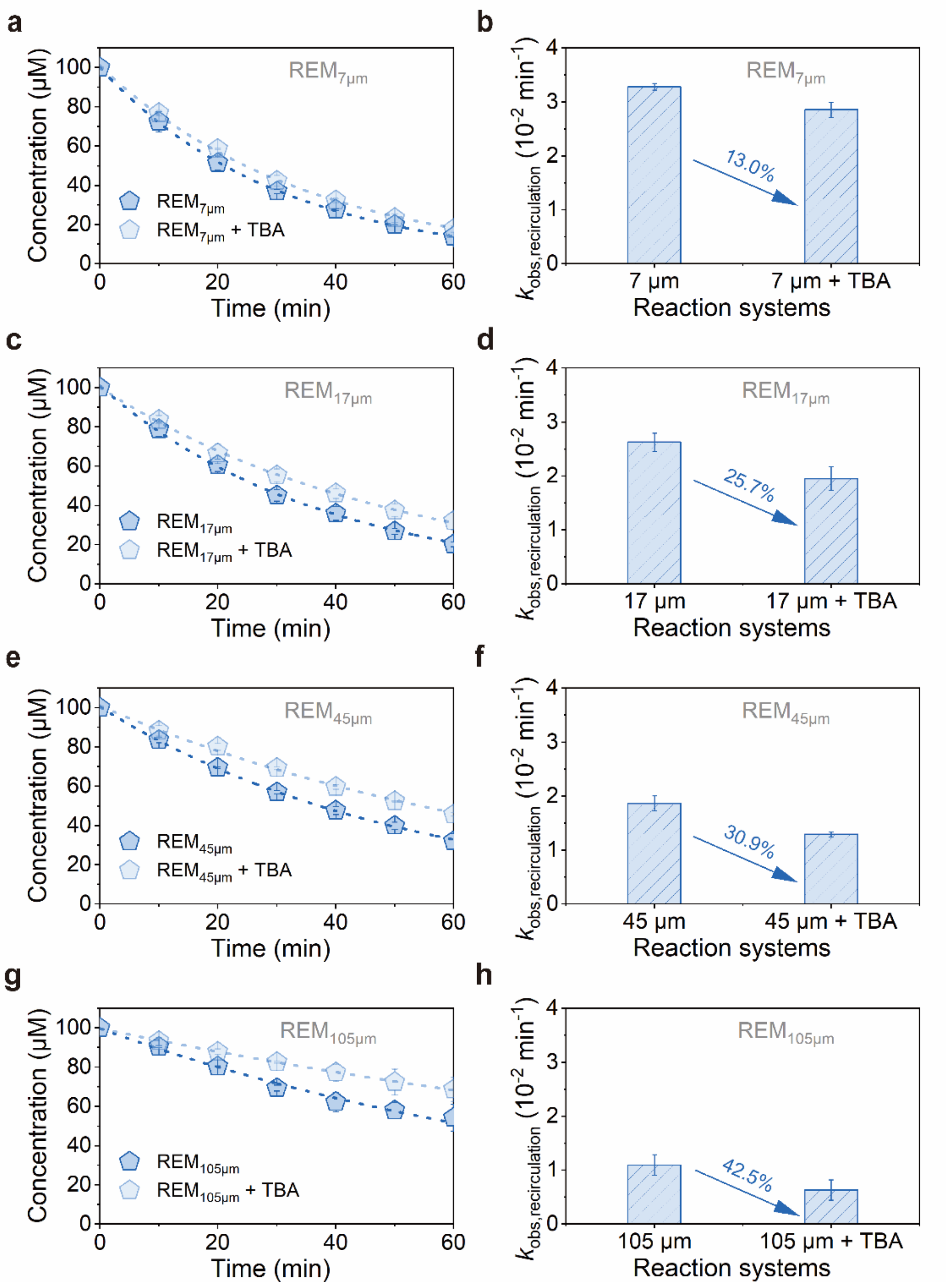

Supplementary Fig. 26. The degradation performance of 4-CP in the recirculation mode. a, c, e, g The effluent 4-CP concentration as a function of HRT on (**a**) $REM_{7μm}$, (**c**) $REM_{17μm}$, (**e**) $REM_{45μm}$, and (**g**) $REM_{105μm}$. **b, d, f, h** The corresponding pseudo-first-order kinetic constants on (**b**) $REM_{7μm}$, (**d**) $REM_{17μm}$, (**f**) $REM_{45μm}$, and (**h**) $REM_{105μm}$. The initial concentration of 4-CP: 100 μM. Current: 19.7 mA cm^{-2}. Volume of water sample: 300 mL. Flux: 0.066 mL cm^{-2} s^{-1}. Error bars represent the data from duplicate tests.

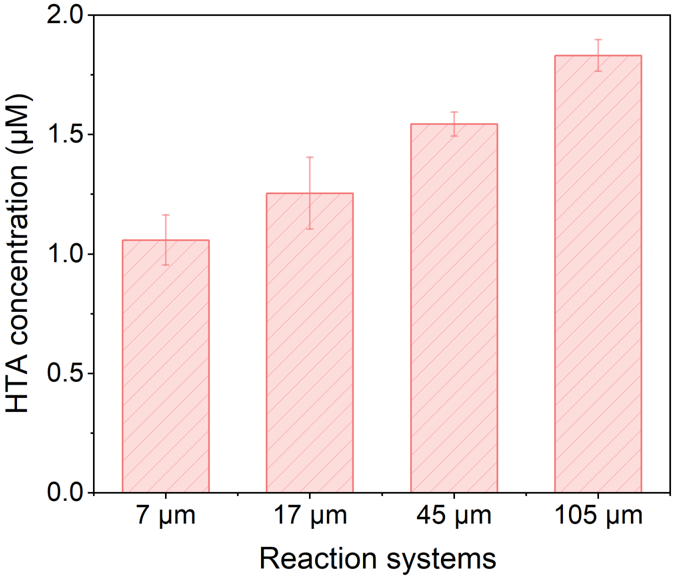

Supplementary Fig. 27. Production of HTA on REMs. HRT: 2.7 s. Potential: 3.8 V_{RHE}. Error bars represent the data from duplicate tests.

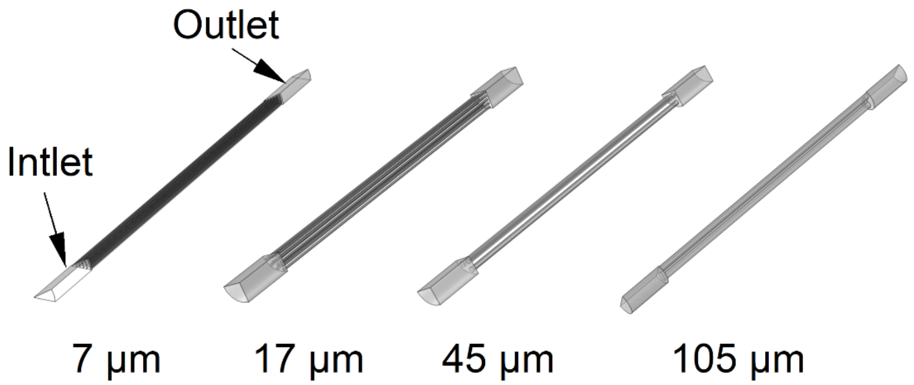

Supplementary Fig. 28. The 3D geometry models of simulated channels. The domain of models has been simplified according to symmetry.

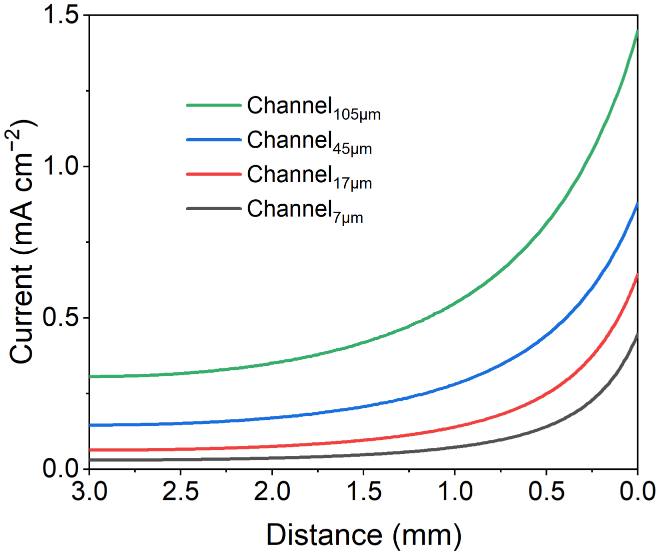

Supplementary Fig. 29. The current distribution of simulated channels along with the distance to the near end.

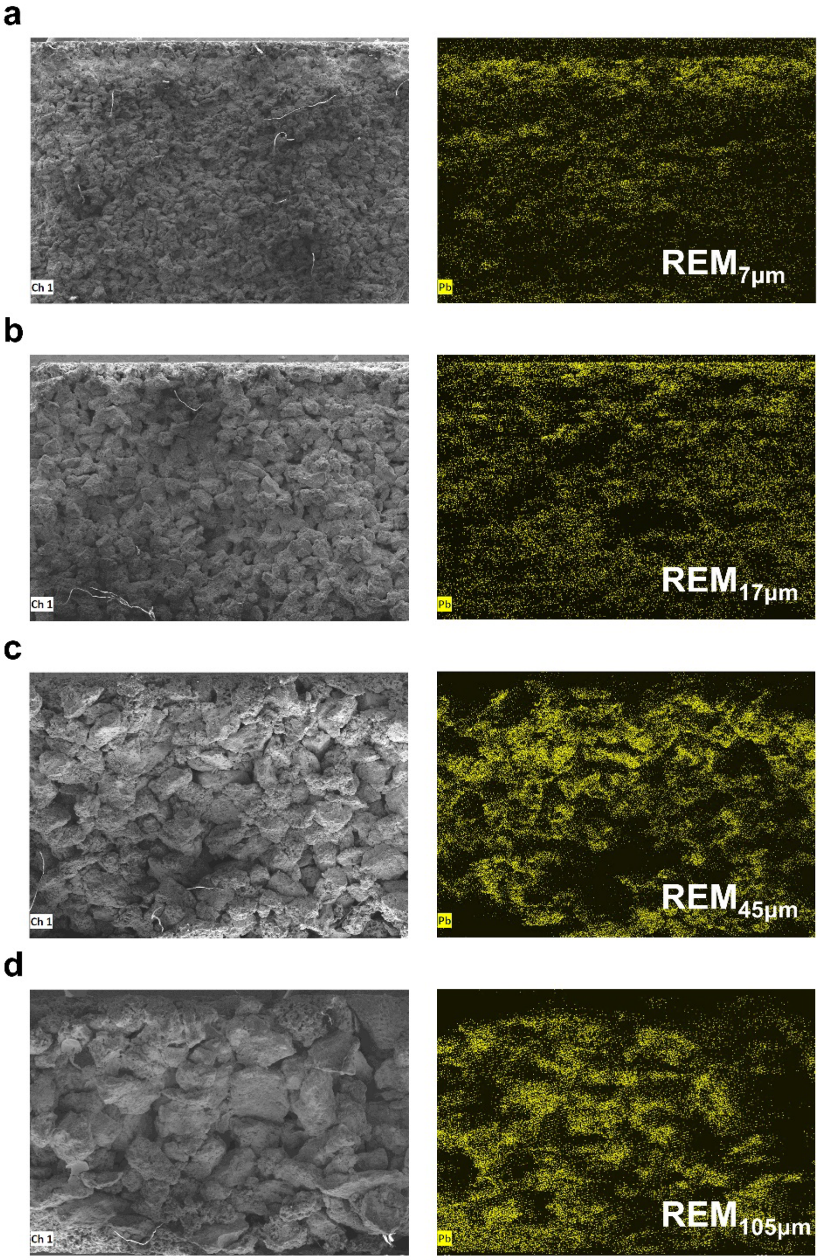

Supplementary Fig. 30. Visualization of the potential distribution in REMs. a-d The cross-sectional SEM image, and Pb elemental mapping on (**a**) $REM_{7\mu m}$, (**b**) $REM_{17\mu m}$, (**c**) $REM_{45\mu m}$, and (**d**) $REM_{105\mu m}$. Scale bars: 800 μm. Current: 19.7 mA cm^{-2}. Reaction time: 60 min. Electrolyte: 0.05 M $Pb(NO_3)_2$, 0.33 M $NaClO_4$.

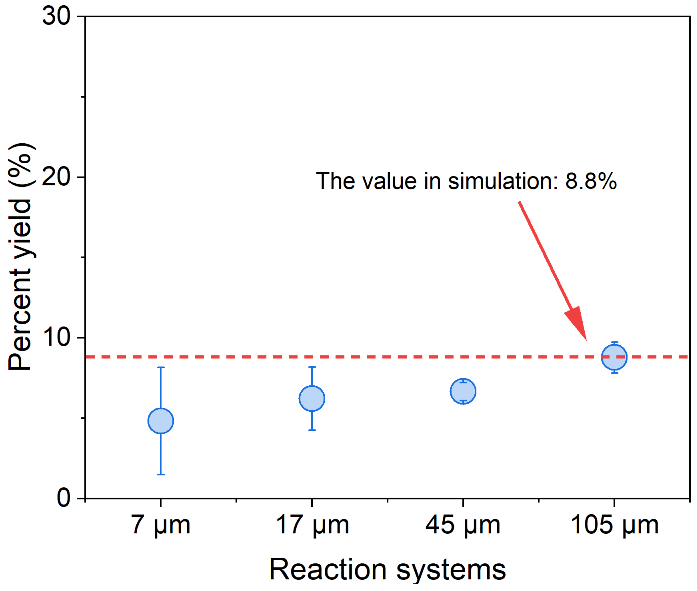

Supplementary Fig. 31. The percent yield of HTA in the experiment. The percent yield is the ratio of HTA production to TA consumption. The initial concentration of TA: 100 μM. Current: 19.7 mA cm^{-2}. HRT: 2.7 s. The percentage yields of HTA obtained on different REMs are slightly different, where the largest pore exhibited the largest yield. This may be attributed to the fact that the oxidation of TA via DET route cannot be neglected, where no HTA is produced in this process[8]. This leads to an underestimation on the percent yield of HTA, especially on $REM_{7μm}$. Therefore, the percentage yield derived from $REM_{105μm}$ (8.8%) was used in the simulation, which is least affected by DET oxidation. Error bars represent the data from duplicate tests.

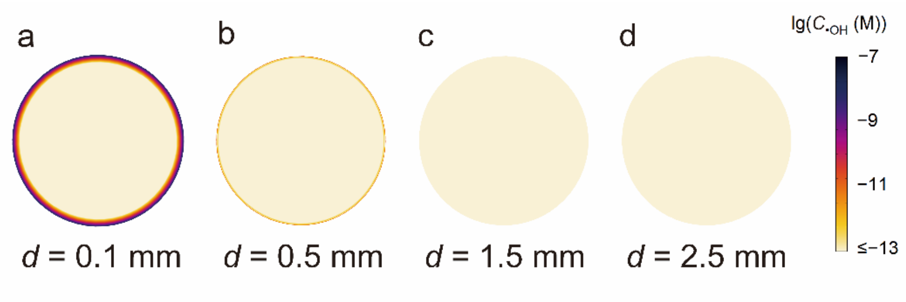

Supplementary Fig. 32. Visualized •OH distribution in the cross-section of channel$_{7\mu m}$. a *d* = 0.1 mm; **b** *d* = 0.5 mm; **c** *d* = 1.5 mm; **d** *d* = 2.5 mm. The thin layer of •OH near the channel surface in panel b was attributed to the diffusion of •OH. HRT: 9.1 s.

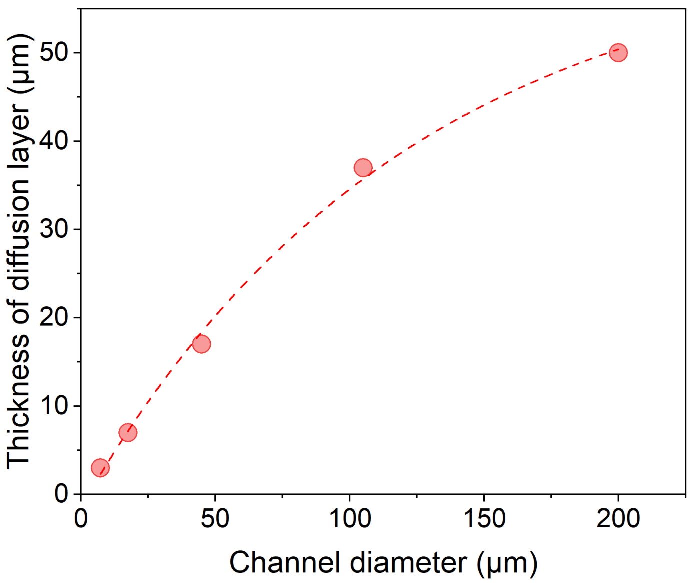

Supplementary Fig. 33. Effect of channel diameter on the thickness of the diffusion layer in the cross-section (*d* = 0.1 mm). The thickness of the diffusion layer of $channel_{200μm}$ was simulated with the same porosity of $channel_{105μm}$. HRT: 9.1 s.

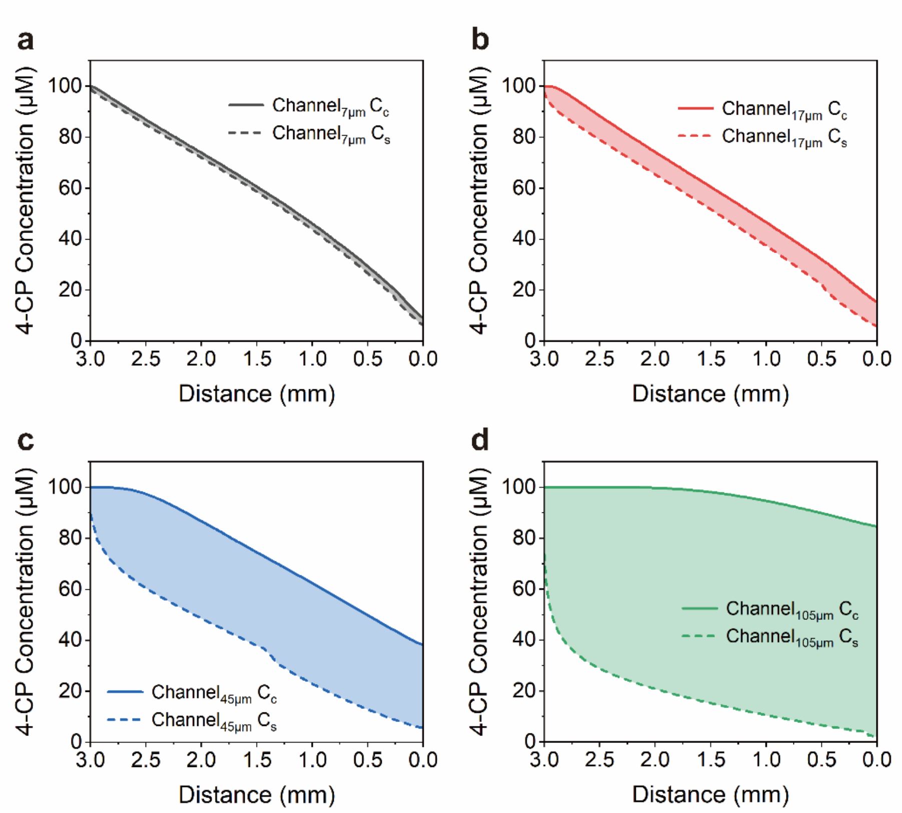

Supplementary Fig. 34. The 4-CP concentration profile. a-d The 4-CP concentration along the flow direction in (**a**) $Channel_{7\mu m}$, (**b**) $Channel_{17\mu m}$, (**c**) $Channel_{45\mu m}$, and (**d**) $Channel_{105\mu m}$. C_c represents 4-CP concentration at the center of the channel. C_s represents 4-CP concentration near the surface of the channel. HRT: 9.1 s.

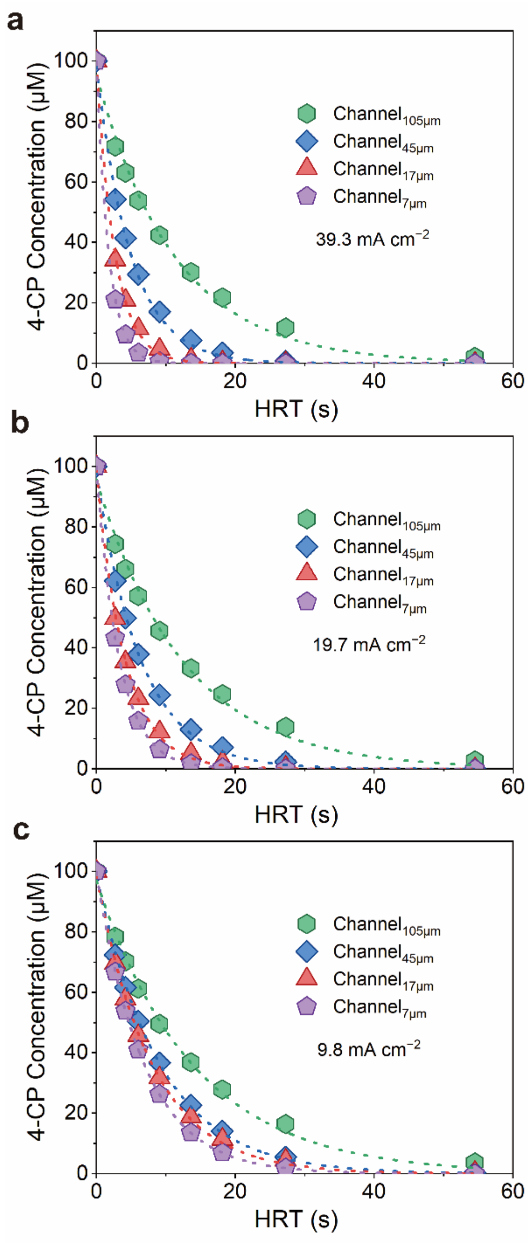

Supplementary Fig. 35. Effluent 4-CP concentration as a function of HRT in different simulated channels. a-c The effluent 4-CP concentration as a function of HRT at current densities of (**a**) 39.3 mA cm^{-2}, (**b**) 19.7 mA cm^{-2}, and (**c**) 9.8 mA cm^{-2}, respectively. The dashed lines represent the fitted degradation curves.

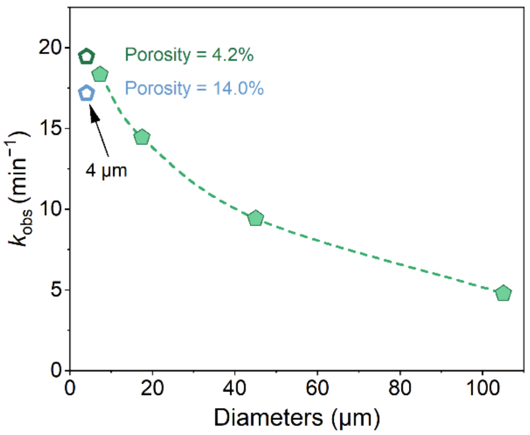

Supplementary Fig. 36. Simulated pseudo-first-order kinetic constants of 4-CP degradation. The simulation of the channel$_{4\mu m}$ was performed with 30% (4.2%) and 100% (14%) of the porosity of the REM$_{7\mu m}$. The electrooxidation performance did not improve when the channel diameter further decreased to 4 μm, due to the fully alleviated concentration polarization. Nonuniform surface potential distribution and the less reactive area also contribute to the slightly lower performance versus channel$_{7\mu m}$. Interestingly, reducing the porosity of the model could evidently increase the reaction kinetics. Although the surface area decreases at relatively low porosity, the surface potential increases, thereby allowing more •OH to be generated.

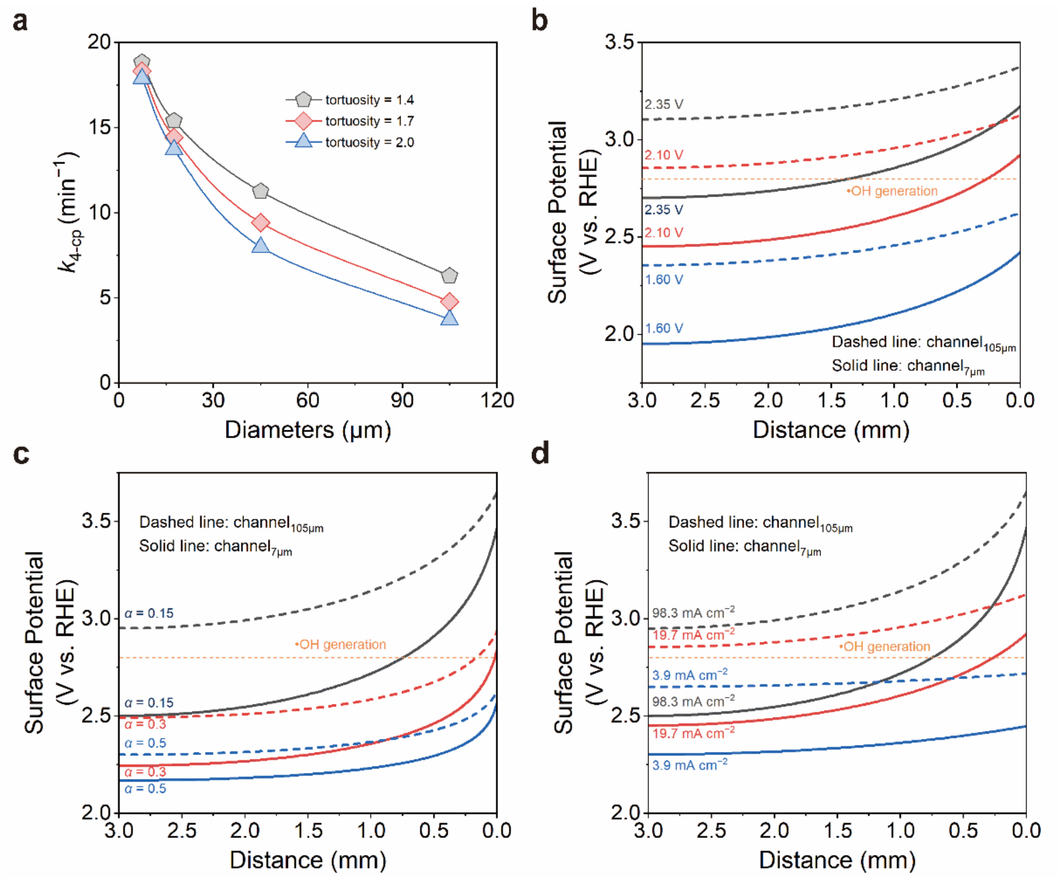

Supplementary Fig. 37. Simulation of the reaction kinetics and potential distribution in the channels. a Simulated reaction kinetics at different tortuosity values. **b** Simulated surface potential distribution in $channel_{105μm}$ and $channel_{7μm}$ at different onset potentials (E_o). **c** Simulated surface potential distribution in $channel_{105μm}$ and $channel_{7μm}$ at different transfer coefficients (α). **d** Simulated surface potential distribution in $channel_{105μm}$ and $channel_{7μm}$ at different applied currents. Due to the slow mass transfer in large channels, the reaction kinetics are greatly affected by tortuosity. The region with •OH-producing activity expands with the increase of E_o. A higher applied current and a larger α lead to a more nonuniform potential distribution. The simulated results follow a similar trend to other studies[25,26].

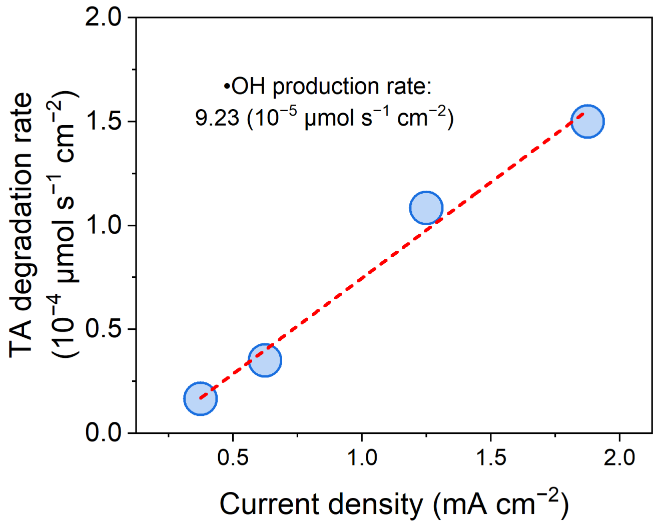

Supplementary Fig. 38. TA degradation rate and •OH production rate on a defective TiO_2 plate electrode. Electrolyte: 0.5 mM TA and 0.33 M $NaClO_4$. The dashed line represents the fitted curve. A defective TiO_2 plate with an area of 8 cm^2 was used as the anode and a Pt wire as the counter electrode. Considering that TA is relatively resistant to DET reaction[8], the reaction with •OH is the dominant oxidation mechanism. Therefore, the •OH production was estimated according to TA degradation. The experiments were conducted at 30 ± 1 °C in 0.33 M $NaClO_4$ with an initial TA concentration of 0.5 mM, which is reported to be high enough to trap all the radicals[27]. The •OH production rate (i.e., the slope of the fitted curve, corresponding to $\frac{\boldsymbol{k}_{\mathbf{g,\bullet OH}}}{\boldsymbol{F}}$ in Supplementary equation (6)) was estimated to be 9.23×10^{-5} µmol cm^{-2} s^{-1} when the current density was 1 mA cm^{-2}. The value of $\boldsymbol{k}_{\mathbf{g,\bullet OH}}$ can be therefore calculated to be 8.91×10^{-6} (C cm^{-2} s^{-1}) and was used in the simulation.

Supplementary Table 1. The surface area of REMs and corresponding microchannels.

	**BET Area**[a] $[m^2\ g^{-1}]$	**Mercury Intrusion Area**[a] $[m^2\ g^{-1}]$	**Cdl**[b] $[F\ cm^{-2}]$
$REM_{7\mu m}$	10.85	9.98	0.41
$REM_{17\mu m}$	6.73	6.59	0.26
$REM_{45\mu m}$	5.36	5.88	0.21
$REM_{105\mu m}$	2.68	2.47	0.10

[a]The Brunauer–Emmett–Teller (BET) surface area and mercury intrusion area reveal the microstructure surface area of REMs and these results show good consistency. The well-recognized BET surface area was used for the subsequent analysis in this study. [b]The C_{dl} value is proportional to the electrochemically active surface area. This result corresponds well with the BET surface area.

Supplementary Table 2. Energy consumption for 4-CP degradation on REMs.[a]

	**Cell Voltage** [V]	***k*** [min^{-1}]	**$HRT_{90\%}$**[b] [s]	**Volume** [cm^3]	**Flow Rate** [10^{-2} mL cm^{-2} s^{-1}]	**ΔP**[c] [bar]	**E_E**[d] [kWh m^{-3}]	**E_P**[e] [kWh m^{-3}]	**Energy Consumption** [kWh m^{-3}]
$REM_{7\mu m}$	4.9	18.9	7.3	0.76	4.09	0.401	0.654	0.209	0.863
$REM_{17\mu m}$	5.1	11.3	12.2	0.76	2.45	0.081	1.137	0.042	1.179
$REM_{45\mu m}$	5.2	7.0	19.7	0.76	1.52	0.022	1.868	0.011	1.879
$REM_{105\mu m}$	5.4	4.6	30.0	0.76	1.00	0.009	2.949	0.005	2.954

[a]The energy consumption was calculated according to the methods described in the Supplementary Text.

[b]$HRT_{90\%}$ refers to the time required to reach 90% 4-CP removal.

[c]ΔP refers to the transmembrane pressure and is determined from Supplementary Fig. 18.

[d]E_E refers to the electrical energy. [e]E_P refers to the pumping energy.

Supplementary Table 3. The porosity of REMs determined by Archimedes' method [28].

	**Average Diameters** [μm]	**Porosity**
REMs	7	14%
	17	19%
	45	26%
	105	32%

REMs were dried overnight and weighed to determine the dry weight. Then, the samples were immersed and sonicated for 30 min to make water fully infiltrate into REMs. After infiltration, the wet weight was measured. Finally, the porosity was calculated using the geometric size of REMs and data collected by the Archimedes' method[28].

Supplementary Table 4. Parameters and values used in the simulation.

Parameters	Value
Oxygen evolution potential[a]	2.1 [V]
Exchange current density of oxygen evolution	4×10^{-2} [A m^{-2}][29]
Electron transferred coefficient for oxygen evolution[b]	0.15
Conductivity of electrolyte	2.54 [S m^{-1}]
Applied current of one channel[c]	0.059, 0.278, 1.203, 5.333 [μA]
Inlet concentration	1×10^{-4} [M]
HRT for 4-CP simulation	2.7 – 54.5 [s]
HRT for TA simulation	2.7 [s]
$k_{g,\bullet OH}$[d]	8.91×10^{-6}
$k_{4\text{-CP},\bullet OH}$	7.6×10^{6} [m^3 s^{-1} mol^{-1}][30]
$k_{TA,\bullet OH}$	4.4×10^{6} [m^3 s^{-1} mol^{-1}][31]
$k_{4\text{-CP},DET}$[e]	0.386 [m^3 mol^{-1}]
$k_{\bullet OH,\bullet OH}$	5.5×10^{6} [m^3 s^{-1} mol^{-1}][32]
$k_{4\text{-CP products},\bullet OH}$[f]	5.0×10^{6} [m^3 s^{-1} mol^{-1}]
•OH diffusion coefficient	2.2×10^{-9} [m^2 s^{-1}][10]
4-CP diffusion coefficient	4.5×10^{-10} [m^2 s^{-1}][33]
TA diffusion coefficient[g]	0.8×10^{-9} [m^2 s^{-1}]
Tortuosity (τ)[h]	1.7[i]

[a]The oxygen evolution potential was determined based on the experiment (Fig. 1c).

[b]The charge transfer coefficient (α) was determined according to other studies. Although a value of $\alpha = 0.5$ is expected on inactive electrodes, several experimental studies have reported $\alpha < 0.5$ for the OER reaction[2,34], even as low as 0.10 for Ti_4O_7[2]. A value of $\alpha = 0.15$ is used in the simulation.

[c]The applied current in representative channels was determined according to the geometric area of electrodes (Supplementary equation (5)).

[d]The rate constant for •OH production was determined based on the experiment (Supplementary Fig. 28).

[e]The rate constant for DET reaction of 4-CP was determined based on the parameters optimization in the simulation.

[f]The rate constant for radical oxidation of intermediate product was determined based on previous studies and parameters optimization in the simulation[30].

[g]The diffusion coefficient of TA was estimated according to other organic molecules (0.67 – 0.99 $m^2 s^{-1}$)[35].

[h]The diffusion coefficient was corrected as $\frac{D}{\tau^2}$[36], because the tortuosity of the pores may inhibit the diffusion process of molecules[37].

[i]The tortuosity was estimated according to a previous study (1.2 – 1.8)[38].

Supplementary References

1 Chen, M. *et al.* Development of a highly efficient electrochemical flow-through anode based on inner in-site enhanced TiO_2-nanotubes array. *Environ. Int.* **140**, 105813 (2020).

2 Khalid, Y. S., Misal, S. N., Mehraeen, S. & Chaplin, B. P. Reactive-transport modeling of electrochemical oxidation of perfluoroalkyl substances in porous flow-through electrodes. *ACS ES. T. Eng.* **2**, 713-725 (2022).

3 Chen, Z., Danilov, D. L., Eichel, R. A. & Notten, P. H. L. Porous Electrode Modeling and its Applications to Li‐Ion Batteries. *Adv. Energy Mater.* **12**, 2201506 (2022).

4 Donaghue, A. & Chaplin, B. P. Effect of select organic compounds on perchlorate formation at boron-doped diamond film anodes. *Environ. Sci. Technol.* **47**, 12391-12399 (2013).

5 Trainham, J. A. & j, N. A flow-through porous electrode model: Application to metal-ion removal from dilute streams. *J. Electrochem. Soc.* **124**, 1528-1540 (1977).

6 Trainham, J. A. & j, N. The effect of electrode placement and finite matrix conductivity on the performance of flow-through porous electrodes. *J. Electrochem. Soc.* **125**, 58-67 (1978).

7 Bard, A. J. & Faulkner, L. R. *Electrochemical Methods: Fundamentals and Applications*. 2nd edn, (Wiley, 2001).

8 Jing, Y. & Chaplin, B. P. Mechanistic study of the validity of using hydroxyl radical probes to characterize electrochemical advanced oxidation processes. *Environ. Sci. Technol.* **51**, 2355-2365 (2017).

9 Yang, K. *et al.* Energy-efficient removal of trace antibiotics from low-conductivity water using a Ti_4O_7 reactive electrochemical ceramic membrane: matrix effects and implications for byproduct formation. *Water Res.* **224**, 119047 (2022).

10 Kapałka, A., Fóti, G. & Comninellis, C. The importance of electrode material in environmental electrochemistry. *Electrochim. Acta.* **54**, 2018-2023 (2009).

11 Marshall, A. T. & Herritsch, A. Understanding how the oxygen evolution reaction kinetics influences electrochemical wastewater oxidation. *Electrochim. Acta.* **282**, 448-458 (2018).

12 Yu, N. *et al.* Electrocatalysis degradation of coal tar wastewater using a novel hydrophobic benzalacetone modified lead dioxide electrode. *Chemosphere* **289**, 133014 (2022).

13 Zheng, J., Wang, Z., Ma, J., Xu, S. & Wu, Z. Development of an electrochemical ceramic membrane filtration system for efficient contaminant removal from waters. *Environ. Sci. Technol.* **52**, 4117-4126 (2018).

14 Sirés, I. *et al.* Electrochemical degradation of paracetamol from water by catalytic action of Fe^{2+}, Cu^{2+}, and UVA light on electrogenerated hydrogen peroxide. *J. Electrochem. Soc.* **153**, D1 (2006).

15 Zhao, G., Zhang, L., Niu, Y. & Sun, K. A molten Mg corrosion method for preparing porous Ti foam as self-supported Li–O_2 battery cathodes. *Electrochim. Acta.* **224**, 64-70 (2017).
16 Gu, Z. *et al.* Interface-modulated nanojunction and microfluidic platform for photoelectrocatalytic chemicals upgrading. *Appl. Catal. B.* **282**, 119541 (2021).
17 Wang, L. *et al.* Blue TiO_2 nanotube electrocatalytic membrane electrode for efficiency electrochemical degradation of organic pollutants. *Chemosphere* **306**, 135628 (2022).
18 Yao, Y. *et al.* Phase change on stainless-steel mesh for promoting sulfate radical formation via peroxymonosulfate oxidation. *Appl. Catal. B.* **278**, 119333 (2020).
19 Lim, J., Yang, Y. & Hoffmann, M. R. Activation of Peroxymonosulfate by Oxygen Vacancies-Enriched Cobalt-Doped Black TiO(2) Nanotubes for the Removal of Organic Pollutants. *Environ. Sci. Technol.* **53**, 6972-6980 (2019).
20 Yang, S. Y., Park, J., Jeong, H. W. & Park, H. Electrocatalytic activities of electrochemically reduced tubular titania arrays loaded with cobalt ions in flow-through processes. *Chem. Eng. J.* **404**, 126410 (2021).
21 Su, T. *et al.* An insight into the role of oxygen vacancy in hydrogenated TiO_2 nanocrystals in the performance of dye-sensitized solar cells. *ACS Appl. Mater. Interfaces* **7**, 3754-3763 (2015).
22 Kim, M. J., Seo, Y., Cruz, M. A. & Wiley, B. J. Metal nanowire felt as a flow-through electrode for high-productivity electrochemistry. *ACS Nano.* **13**, 6998-7009 (2019).
23 Arenas, L. F., Ponce de León, C. & Walsh, F. C. 3D-printed porous electrodes for advanced electrochemical flow reactors: A Ni/stainless steel electrode and its mass transport characteristics. *Electrochem. Commun.* **77**, 133-137 (2017).
24 Chen, L. *et al.* Accurate identification of radicals by in-situ electron paramagnetic resonance in ultraviolet-based homogenous advanced oxidation processes. *Water Res.* **221**, 118747 (2022).
25 Lasia, A. Impedance of porous electrodes. *J. Electroanal. Chem.* **397**, 27-33 (1995).
26 Yang, K., Zu, D., Zhang, Z., Ma, J. & Yang, Z. Mechanistic Insight into the Spatial Scale of Nonuniform Oxidation of Micropollutants in Reactive Electrochemical Membranes for Water Purification. *ACS ES. T. Eng.* (2023).
27 Ishibashi, K.-i., Fujishima, A., Watanabe, T. & Hashimoto, K. Detection of active oxidative species in TiO_2 photocatalysis using the fluorescence technique. *Electrochem. Commun.* **2**, 207-210 (2000).
28 Chen, C. *et al.* High performance of anode supported $BaZr_{0.1}Ce_{0.7}Y_{0.1}Yb_{0.1}O_{3-\delta}$ proton-conducting electrolyte micro-tubular cells with asymmetric structure for IT-SOFCs. *J. Electroanal. Chem.* **844**, 49-57 (2019).
29 You, S. *et al.* Monolithic Porous Magnéli-phase Ti4O7 for Electro-oxidation Treatment of Industrial Wastewater. *Electrochim. Acta.* **214**, 326-335 (2016).

30 Wang, Y.-n., Chen, J., Li, X., Zhang, S. & Qiao, X. Estimation of Aqueous-Phase Reaction Rate Constants of Hydroxyl Radical with Phenols, Alkanes and Alcohols. *QSAR Comb. Sci.* **28**, 1309-1316 (2009).

31 Pei, S., You, S., Ma, J., Chen, X. & Ren, N. Electron spin resonance evidence for electro-generated hydroxyl radicals. *Environ. Sci. Technol.* **54**, 13333-13343 (2020).

32 Li, X. *et al.* Electro-catazone treatment of ozone-resistant drug ibuprofen: Interfacial reaction kinetics, influencing mechanisms, and degradation sites. *J. Hazard. Mater.* **4**, 100023 (2021).

33 Martins, L. F. G., Parreira, M. C. B., Ramalho, J. P. P., Morgado, P. & Filipe, E. J. M. Prediction of diffusion coefficients of chlorophenols in water by computer simulation. *Fluid Phase Equilib.* **396**, 9-19 (2015).

34 Kapałka, A., Fóti, G. & Comninellis, C. Determination of the Tafel slope for oxygen evolution on boron-doped diamond electrodes. *Electrochem. Commun.* **10**, 607-610 (2008).

35 Wen, W., Zhao, H., Zhang, S. & Pires, V. Rapid Photoelectrochemical Method for in Situ Determination of Effective Diffusion Coefficient of Organic Compounds. *J. Phys. Chem. C* **112**, 3875-3880 (2008).

36 Misal, S. N., Lin, M. H., Mehraeen, S. & Chaplin, B. P. Modeling electrochemical oxidation and reduction of sulfamethoxazole using electrocatalytic reactive electrochemical membranes. *J. Hazard. Mater.* **384**, 121420 (2020).

37 Petersen, E. E. Diffusion in a pore of varying cross section. *AICHE J.* **4**, 343-345 (1958).

38 Chen-Wiegart, Y.-c. K. *et al.* 3D morphological evolution of porous titanium by x-ray micro- and nano-tomography. *J. Mater. Res.* **28**, 2444-2452 (2013).
