# Supplementary material for: Unveiling the spatially confined oxidation processes in reactive electrochemical membranes
Source: Nat Commun. 2023 Oct 18;14:6590. doi: 10.1038/s41467-023-42224-3 (PMC10584896; doi:10.1038/s41467-023-42224-3)
Supplement: Supplementary file 3 — Description of Additional Supplementary Files [file 41467_2023_42224_MOESM3_ESM.pdf]

Description of Additional Supplementary Files

File Name: Supplementary Movie 1
Description: Visualized 4-CP distribution in simulated channels
