# Supplementary figures and images for: Unveiling the spatially confined oxidation processes in reactive electrochemical membranes
Source: Nat Commun. 2023 Oct 18;14:6590. doi: 10.1038/s41467-023-42224-3 (PMC10584896; doi:10.1038/s41467-023-42224-3)

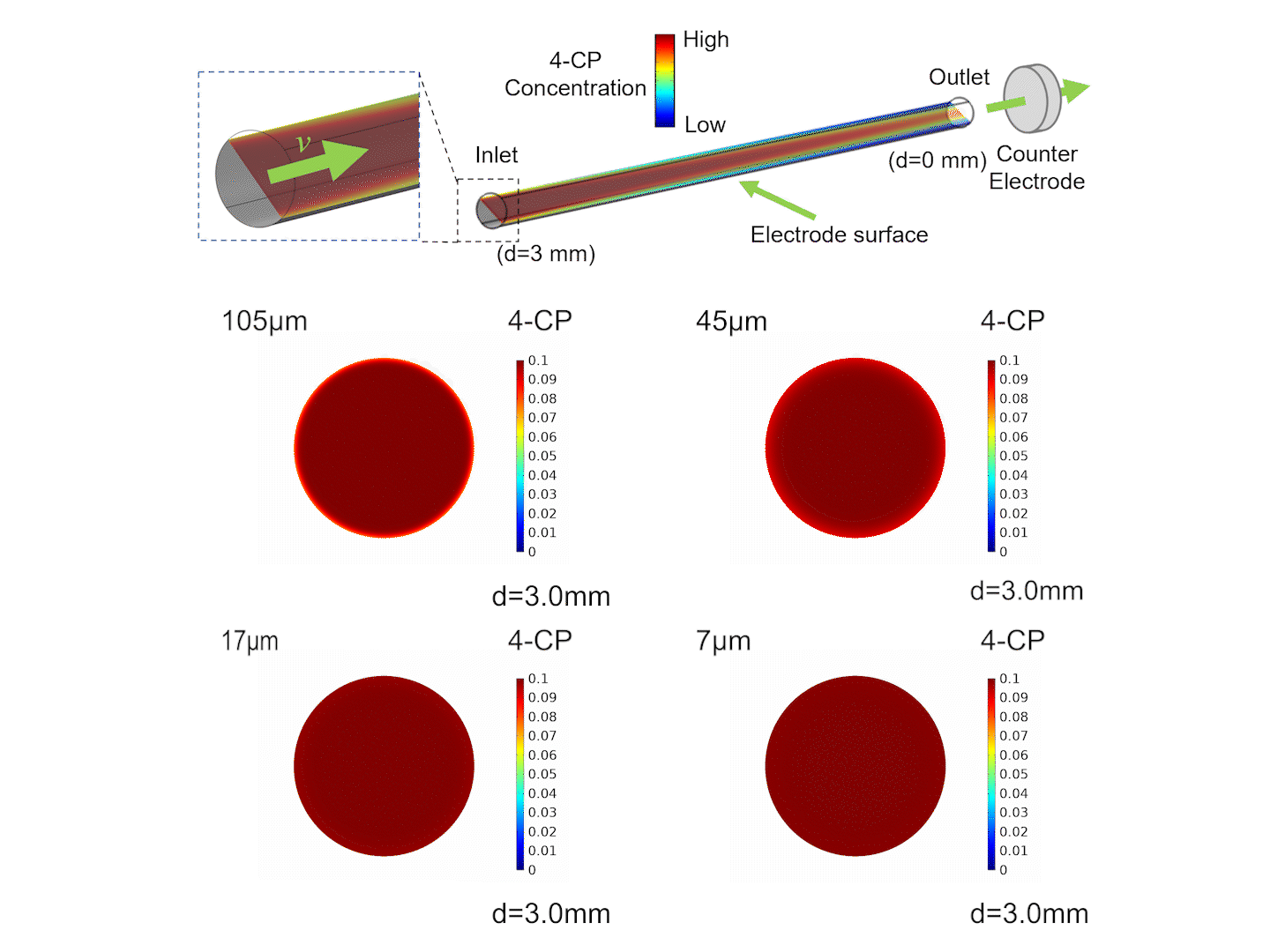

Supplement: Supplementary file 4 — Supplementary Movie 1 [file 41467_2023_42224_MOESM4_ESM.gif]
